# A *Zap70* gain-of-function mutation alters mouse thymic maturation

**DOI:** 10.3389/fimmu.2026.1932213

**Published:** 2026-10-06

**Authors:** Inmaculada Vico-Barranco, Fanghui Zhang, Claude Grégoire, Luis M. Fernández-Aguilar, Mikel M. Arbulo-Echevarria, Isaac Narbona-Sánchez, Raquel Muñoz-Fernández, Bernard Malissen, Marie Malissen, Enrique Aguado

**Affiliations:** 1 Department of Biomedicine, Biotechnology and Public Health, Universidad de Cádiz, Cádiz, Spain; 2 Centre d’Immunophénomique (CIPHE), Aix Marseille Université, INSERM, CNRS, Marseille, France; 3 Centre d’Immunologie de Marseille-Luminy (CIML), Aix Marseille Université, INSERM, CNRS, Marseille, France; 4 School of Medical Technology, Xinxiang Medical University, Xinxiang, Henan, China; 5 Institute of Biomedical Research Cadiz (INIBICA), Cádiz, Spain; 6 Laboratory of Immunophenomics, School of Medical Technology, Xinxiang Medical University, Xinxiang, Henan, China

**Keywords:** LCK, TCR signaling, thymic development, Treg, ZAP-70

## Abstract

The tyrosine kinase ZAP-70 is essential for T-cell activation and development, but the mechanisms regulating its activity remain unclear. Previous studies showed that the Src-family kinase LCK binds phosphorylated tyrosine 319 of ZAP-70 via its SH2 domain. Additionally, a gain-of-function ZAP-70 mutation involving the YEEI motif enhances LCK recruitment and TCR-mediated signaling. To investigate the physiological consequences of enhanced ZAP-70-LCK interaction *in vivo*, we generated a knock-in mouse model expressing the *Zap70*^YEEI^ gain-of-function mutation. Analysis of thymocyte development revealed that the *Zap70*^YEEI^ mutation altered T-cell maturation, resulting in increased differentiation of thymic regulatory T (Treg) cells and reduced thymic negative selection. In the peripheral T-cell compartment, mutant mice exhibited decreased surface expression of the TCRαβ complex and an increased frequency of memory CD4 T cells. These findings indicate that enhancing the ZAP-70-LCK interaction modifies signaling thresholds during thymic selection and peripheral T-cell homeostasis. Collectively, our results demonstrate that a gain-of-function mutation in *Zap70* significantly reshapes T-cell development and maturation, highlighting the importance of regulated ZAP-70-LCK interactions in establishing immune tolerance and maintaining T-cell compartment balance.

## Introduction

Host defense against malignancies and pathogens relies heavily on T cell-mediated immunity. The hallmark of this system is its antigen-specific recognition, which facilitates a potent yet self-controlled immune response across a vast array of challenges. Upon the engagement of the T cell receptor (TCR), a precisely orchestrated sequence of intracellular signaling pathways is initiated, driving the activation, expansion, and functional maturation of T cells. Furthermore, these signaling mechanisms are vital during T cell ontogeny, ensuring the production of a protective repertoire while filtering out self-reactive T cells.

Stimulation of the TCR on T cells rapidly leads to the phosphorylation of the tyrosine residues of the Immunoreceptor Tyrosine-based Activation Motifs (ITAMs) located in the cytoplasmic segments of the associated CD3 chains (1, 2). This phosphorylation event is the first biochemical reaction to occur following antigen recognition by T cells and is carried out by the lymphocyte-specific protein tyrosine kinase (LCK) (3, 4). ITAMs phosphorylation generates docking sites for the ζ chain-associated protein kinase 70 (ZAP-70) that facilitate downstream signal transduction (5). ZAP-70 recruitment to dually phosphorylated ITAMs triggers a conformational shift, realigning its SH2 domains and disrupting autoinhibitory constraints in the linker region (6). This structural transition primes ZAP-70 for LCK-mediated phosphorylation, which is necessary to achieve its full catalytic activation (7, 8). Once activated, ZAP-70 phosphorylates tyrosine residues on the adaptor proteins LAT and SLP-76 (1, 9). This event coordinates the assembly of a multiprotein signalosome, which bridges the TCR-CD3 complex to the primary downstream signaling pathways required for T cell activation.

ZAP-70 belongs to the Syk family of protein tyrosine kinases, which includes only SYK and ZAP-70. Both kinases show different expression patterns, with Syk being expressed by B cells, macrophages, monocytes and platelets, and ZAP-70 mainly restricted to T cells and NK cells (1, 5). ZAP-70 comprises two tandem SH2 domains and a carboxy-terminal kinase domain. An unstructured region, referred to as interdomain B, connects the SH2 domains to the kinase domain. In the mouse, this region contains three tyrosine residues at position 292, 315 and 319 that are phosphorylated upon TCR activation and participate in the regulation of ZAP-70 enzymatic activity. The human ZAP-70 kinase domain contains two tyrosine residues at positions 492 and 493. Located within the activation loop, the phosphorylation of these sites is essential for the induction of enzymatic activity (6).

Underscoring its clinical relevance, aberrant ZAP-70 signaling output is a major driver of human pathology. While a complete loss of ZAP-70 function abolishes T-cell responses and causes severe combined immunodeficiency (SCID) (10), its ectopic expression in B cells serves as a poor prognostic marker in chronic lymphocytic leukemia (11). Conversely, mutations that subtly augment ZAP-70 signaling disrupt tolerance mechanisms and predispose to autoimmunity (12). These clinical phenotypes emphasize that immune homeostasis is governed by the precise quantitative tuning of ZAP-70 signaling output.

The physiological importance of tyrosine residues located within interdomain B is underscored by the severe defects in thymic development observed in mouse models when they are mutated to phenylalanine (13, 14). Y315F and Y319F substitutions attenuate both positive and negative selection, whereas the Y292F mutation exerts an opposing effect by upregulating proximal TCR signaling. Further analysis using Jurkat cells clarified the mode of action of those mutations; the Y319F mutation (*Zap70*^Y319F^) specifically and drastically reduced the tyrosine phosphorylation of LAT and SLP-76 adaptors, thereby impairing downstream NFAT activation and IL-2 production (15). Y319 is part of a YSDP sequence and constitutes a consensual SH2-binding motif. However, this YSDP motif exhibits a lower affinity for the LCK-SH2 domain than an optimized engineered YEEI sequence (16). Replacing in Jurkat cells the Y_319_SDP sequence by the Y_319_EEI sequence resulted in a mutant allele that is denoted *Zap70*^YEEI^ and that significantly amplified TCR-associated signaling. Because this gain-of-function mutation kept an enzymatic activity and phospho-ITAM binding affinity comparable to wild-type ZAP-70, these findings reveal that the interaction between Y319 and the LCK SH2 domain plays a pivotal role for propagating the TCR signaling cascade and that enhancing it via the *Zap70*^YEEI^ mutation suffices to the amplify TCR signaling (16).

Consistent with these *in vitro* findings, studies using a transgenic mouse model expressing the ZAP-70 gain-of-function mutant (*Zap70*^YEEI^ Tg) demonstrated that CD8^+^ T cells exhibit elevated basal phosphorylation of LAT and Erk, as well as enhanced signaling 30 seconds post-stimulation with anti-CD3 antibodies (17). Conversely, at later time points, both TCR-dependent intracellular signaling and IFN-γ production were diminished in these cells, suggesting the potentiation of downstream negative feedback mechanisms. Combined with data from ZAP-70-deficient Jurkat cells (18), these results support a model in which LCK-mediated phosphorylation of Y319 serves a dual role: it recruits both activatory and inhibitory effector molecules while simultaneously relieving intrinsic autoinhibition to enable full kinase activity. Considering that the above study is focused exclusively on CD8^+^ T cells, and that the transgene coding for *Zap70*^YEEI^ is only expressed from the CD8SP stage onward it prevented the analysis of its effects on other key events in thymic development. Therefore, to more thoroughly investigate the role of the ZAP-70-LCK interaction in T cell development and activation, we generated a knock-in mouse model harboring the YEEI motif within the interdomain B of mouse ZAP-70 (*Zap70*^YEEI^ knock-in mice). While homozygous mutant mice were born at expected Mendelian frequencies and exhibited normal survival, they displayed significant alterations in T cell homeostasis and selection. Our results demonstrate that the enhanced ZAP-70-LCK interaction promotes the differentiation of thymic regulatory T (Treg) cells and impairs thymic negative selection. Furthermore, we observed reduced levels of surface TCRαβ expression on peripheral T cells alongside a significant expansion of the memory CD4^+^ T lymphocyte compartment, suggesting that the *Zap70*^YEEI^ mutation fundamentally recalibrates the threshold for T cell signaling and peripheral maintenance. These results establish that the ZAP-70-LCK interaction is an essential determinant of T cell fate, demonstrating that subtle modifications to this signaling node can profoundly alter the landscape of the adaptive immune system.

## Results

### Generation of mutant mice

Mice carrying the Y_319_SDP to Y_319_EEI mutation and denoted *Zap70*^YEEI^ were generated using a gene-editing approach similar to the one we formerly used to generate mice that express ZAP-70 molecules with Tyr to Phe substitution at position 292 or 315 (**Supplementary Figure 1**). This approach ensures the expression of ZAP-70 mutant molecules at physiological levels and with appropriate developmental kinetics. Germline transmission was achieved by crossing chimeric mice derived from a validated embryonic stem cell clone with *Cre*-expressing deleter mice (19) to delete the loxP-flanked neomycin resistance cassette. The resulting progeny were backcrossed onto a C57BL/6 background prior to subsequent experiments. Due to the high degree of sequence conservation between human and mouse ZAP-70 interdomains B, we kept the human tyrosine numbering for the mouse protein to facilitate direct interspecies comparison. Homozygous and heterozygous mutant mice were born at the expected Mendelian frequencies (not shown and **Supplementary Figure 2A**), and their viability similar to wild-type littermates. To rule out potential confounding effects of age-related thymic involution across groups, analyses were restricted to 6–16-week-old mice, and total thymocyte cellularity, along with sex and exact age, was individually cataloged for all mice analyzed (**Supplementary Table 1**). Within this age window, thymocyte yields remained stable across ages in both male and female mice. ZAP-70 protein expression in thymocytes from homozygous mutant mice was comparable to levels observed in wild-type mice (**Supplementary Figure 2D**).

### *Zap70*^YEEI^ mutation does not grossly impair thymocyte development

We first determined the impact of the *Zap70*^YEEI^ mutation on T-cell development. Total thymic cellularity of both heterozygous and homozygous *Zap70*^YEEI^ mice was found indistinguishable from that of wild-type littermates (**Supplementary Figure 2B**). To focus with a high-resolution on T-cell precursors, we used a “dump” channel (comprising MHC-II, CD11b, and NK1.1) that permit to exclude contaminating antigen-presenting, myeloid, and NK cells (**Supplementary Figure 3**). Analysis of CD4 and CD8 expression profiles revealed that the relative proportions of double-negative (DN, CD4^−^CD8^−^), double-positive (DP, CD4^+^CD8^+^), and single-positive (CD4SP and CD8SP) compartments in heterozygous and homozygous *Zap70*^YEEI^ mice were comparable to wild-type mice (**Figure 1A**). Furthermore, the commitment to the γδ lineage remained unaffected, as evidenced by the presence of similar percentages and numbers of TCRγδ^+^ thymocytes in mutant and wild-type mice (**Supplementary Figure 4A**).

**Figure 1:**
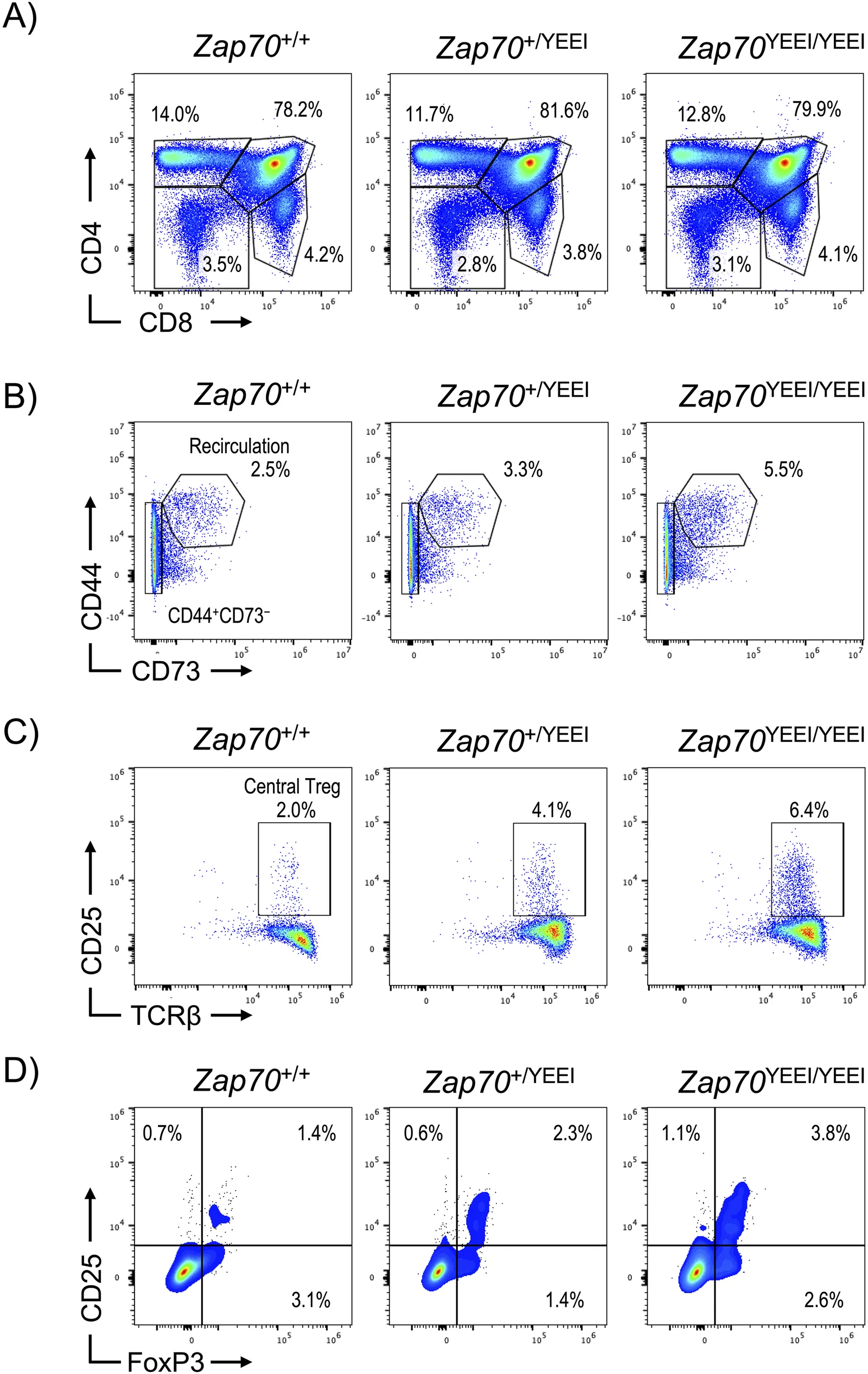
Flow cytometric analysis of thymic development. **(A)** Thymuses from 6- to 16-week-old adult wild-type (*Zap70*^+/+^), heterozygous (*Zap70*^+/YEEI^), and homozygous (*Zap70*^YEEI/YEEI^) mutant mice were disaggregated and live thymocytes were analyzed by flow cytometry using the gating strategy shown in **Supplementary Figure 3**. TCRγδ^−^ dump^−^ thymocytes were stained with antibodies specific for CD4 and CD8. Numbers within gates indicate the percentage of cells in each subset. One representative experiment is shown. **(B)** Recirculating thymocytes were assessed based on CD44 and CD73 expression. One representative experiment is shown. **(C)** The Treg compartment was analyzed within the CD44^+^CD73^−^ population according to TCRβ and CD25 expression. **(D)** Dot plots show FoxP3 and CD25 expression in CD4 single-positive thymocytes from *Zap70*^+/+^, *Zap70*^+/YEEI^, and *Zap70*^YEEI/YEEI^ mice. One representative experiment is shown.

We next focused on the early stages of thymic differentiation following the gating strategy outlined in **Supplementary Figure 3**. The distribution of DN1 to DN4 cell stages was equivalent between wild-type and mutant mice (**Supplementary Figure 4B**). We specifically focused on the transition from the DN3a to the DN3b stage, a critical checkpoint characterized by the concurrent rearrangement of the *Trb*, *Trg*, and *Trd* loci and the subsequent commitment to either the αβ or γδ lineage (20). Consistent with our analysis of DN, DP and SP cell stages, the DN3a/DN3b ratio was maintained in both heterozygous and homozygous mutants (**Supplementary Figure 4C**).

Finally, we examined the maturation of CD8SP cells. The proportions of intermediate CD8 single positive cells (characterized as TCRβ^lo^CD24^hi^) as well as mature TCRβ^hi^CD24^lo^ populations, showed no significant deviations from wild-type controls (**Supplementary Figures 5D, E**). Collectively, these data demonstrate that the *Zap70*^YEEI^ mutation does not exert a detectable effect on the establishment of the major thymic populations or the fundamental checkpoints of T-cell development.

### *Zap70*^YEEI^ mutation promotes the expansion of thymic regulatory T-cell precursors

Since thymic regulatory T (Treg) cell development is positively correlated with TCR affinity and signaling strength (21, 22), we investigated whether the *Zap70*^YEEI^ mutation (which is expected to augment TCR-associated signaling) impacts Treg cell differentiation. To accurately assess *de novo* Treg development, we first distinguished resident thymic populations from recirculating mature cells (**Supplementary Figure 3**). Given that CD73 has been identified as a reliable marker for peripheral Treg cells that have re-entered the thymus (23, 24), and that nascent CD4SP thymocytes express low-to-intermediate levels of CD44, we analyzed the expression of CD44 and CD73 on CD4SP thymocytes. Notably, homozygous mutant mice exhibited a statistically significant increase in the total number of CD44^+^CD73^+^ recirculating CD4SP cells compared to wild-type controls (**Figure 1B**; **Supplementary Figure 5A**, left panel) confirming an altered migratory or homeostatic profile in the mutant thymus.

We subsequently focused our analysis on the non-recirculating (CD44^lo^CD73^−^) CD4SP population. Notably, we detected a significant increase in the percentage of CD25 expression in both heterozygous (*Zap70*^+/YEEI^) and homozygous (*Zap70*^YEEI/YEEI^) mutant mice compared to wild-type controls, suggesting an expansion of the Treg cell precursor pool (**Figure 1C**). This expansion appeared to be gene-dosage-dependent, as the increase in heterozygous mice was less pronounced than that observed in homozygous littermates (**Supplementary Figure 5A**, bottom panel). This observation was further corroborated by the expression of the lineage-specifying transcription factor Foxp3; the proportion of Foxp3^+^ cells was significantly elevated in both homozygous and heterozygous mutant mice relative to wild-type counterparts (**Figure 1D**).

Finally, we characterized the terminal maturation of Foxp3-negative T cells that represent the conventional CD4 lineage. By examining the expression of CD62L and CD24 within the CD4^+^TCRβ^+^CD25^−^ compartment (**Supplementary Figure 3**), we were able to discriminate between immature (CD24^hi^CD62L^lo^) and mature (CD24^lo^CD62L^hi^) thymocytes. As shown in **Supplementary Figures 5B, C**, the *Zap70*^YEEI^ mutation had no detectable effect on these final maturation steps. Collectively, these results indicate that while the *Zap70*^YEEI^ mutation does not alter the general maturation of conventional CD4SP thymocytes, it specifically enhances the differentiation and expansion of the thymic Treg cell lineage.

### The *Zap70*^YEEI^ mutation attenuates clonal deletion and death by neglect

We next investigated the impact of the *Zap70*^YEEI^ mutation on the thymic selection processes. Mature αβ TCRs are first expressed during the DP stage, where their individual capability to recognize self-peptide-MHC (pMHC) complexes can be evaluated by the surface expression of CD5 and TCRβ on individual T cells (25, 26). Thymocytes expressing high levels of both markers (TCRβ^hi^CD5^hi^) are considered “signaled” in that they have successfully undergone positive selection via intermediate-affinity interactions with self-pMHC. Within this signaled T cell population, those that bind self-antigens with excessively high affinity are eliminated by negative selection (clonal deletion), a process that can be monitored by the intracellular expression of active caspase 3 (**Figure 2A**) (26). Conversely, cells with low CD5 expression represent “non signaled” thymocytes that fail to recognize self-pMHC and are consequently eliminated by “death by neglect” (27), which can also be identified by the expression of active caspase 3.

**Figure 2:**
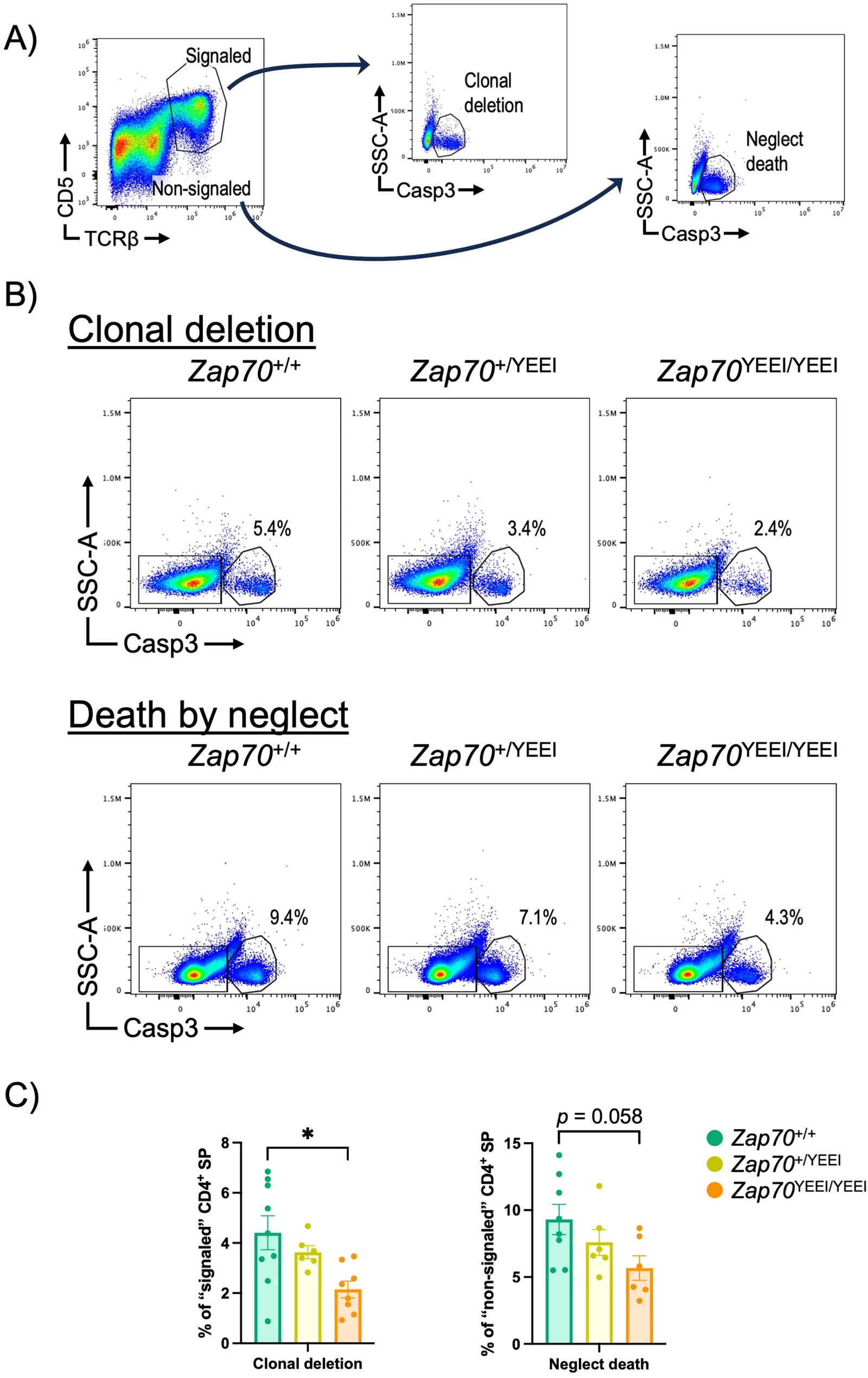
The *Zap70*^YEEI^ mutation disturbs thymic selection. **(A)** Flow cytometry gating strategy for the identification of cells undergoing clonal deletion or death by neglect in the thymus. Signaled and non-signaled thymocytes identified by the expression of CD5 and TCRβ, excluding MHC-II^+^, CD11b^+^, and NK1.1^+^ (Dump) cells. Clonal deletion and death by neglect were identified by the expression of cleaved caspase 3. **(B)** Dot plots showing clonal deletion (upper panels) and death by neglect (bottom panels) in thymuses from adult wild-type (*Zap70*^+/+^), heterozygous (*Zap70*^+/YEEI^), and homozygous (*Zap70*^YEEI/YEEI^) mice. Numbers next to the gates indicate the percentage of cells in each subset. One representative experiment is shown. **(C)** Bar graphs showing the mean percentages of clonal deletion in signaled cells (left panel) and death by neglect in non-signaled thymocytes (right panel) in the thymus of wild-type (n = 8), heterozygous (n = 6, and homozygous (n = 6) mutant mice, aged from 6 to 16 weeks. Brackets on each bar represent the standard error of the mean. Asterisks represent statistical significance (*p < 0.05).

Intriguingly, when expressed as homozygous and heterozygous states, the *Zap70*^YEEI^ mutation resulted in a significant decrease in clonal deletion as compared to wild-type mice (**Figures 2B, C**). In heterozygous *Zap70*^+/YEEI^ mice, the frequency of negatively selected cells was intermediate between wild-type and homozygous mutant values, though this trend did not reach statistical significance. Furthermore, thymocytes undergoing death by neglect displayed a downward trend in homozygous *Zap70*^YEEI/YEEI^ mutant mice compared to wild-type controls, closely approaching significance (*p* = 0.058). These findings demonstrate that the *Zap70*^YEEI^ mutation primary dampens negative selection, alongside a subtle reduction in death by neglect during thymic maturation.

### TCR-signaling is augmented in *Zap70*^YEEI^ mutant thymocytes

Previous *in vitro* studies have demonstrated that ZAP-70^YEEI^ mutant molecules have an increased ability to bind LCK, thereby inducing elevated TCR-associated signaling (16, 17). To investigate whether the ZAP-70^YEEI^ mutant molecules expressed by our knock-in mouse model displayed the same functional characteristics, we analyzed TCR-induced intracellular signals in thymocytes from heterozygous (*Zap70^+/^*^YEEI^) and homozygous (*Zap70^YEEI/^*^YEEI^) mutant mice following CD3 crosslinking. Freshly isolated thymocytes were incubated with biotin-conjugated anti-CD3 antibodies (5 μg/ml) and subsequently stimulated with streptavidin at 37°C for 0, 3, 10, and 30 minutes. Consistent with established signaling kinetics, anti-CD3 stimulation in wild-type (*Zap70*^+/+^) thymocytes induced a peak in phosphorylation of residue Y136 of LAT (LAT-Y136) at 3 minutes, which returned to baseline by 30 minutes (**Figure 3A**). Notably, the magnitude of LAT-Y136 phosphorylation was enhanced in *Zap70^+/^*^YEEI^ and *Zap70^YEEI/^*^YEEI^ mutant mice, with statistically significant differences between *Zap70*^+/+^ and *Zap70^YEEI/^*^YEEI^ mice at 3 min. This augmentation followed a dose-dependent pattern, with heterozygous cells exhibiting intermediate phosphorylation levels between wild-type and homozygous mutant animals (**Figure 3B**). Parallel analysis of LAT-Y195 phosphorylation revealed an identical trend, further confirming the hyper-responsive nature of the mutant thymocytes. Importantly, analysis of total ZAP-70 protein levels confirmed that this observed increase in TCR signaling was not attributable to elevated ZAP-70 expression (**Figure 3A**; **Supplementary Figure 2D**).

**Figure 3:**
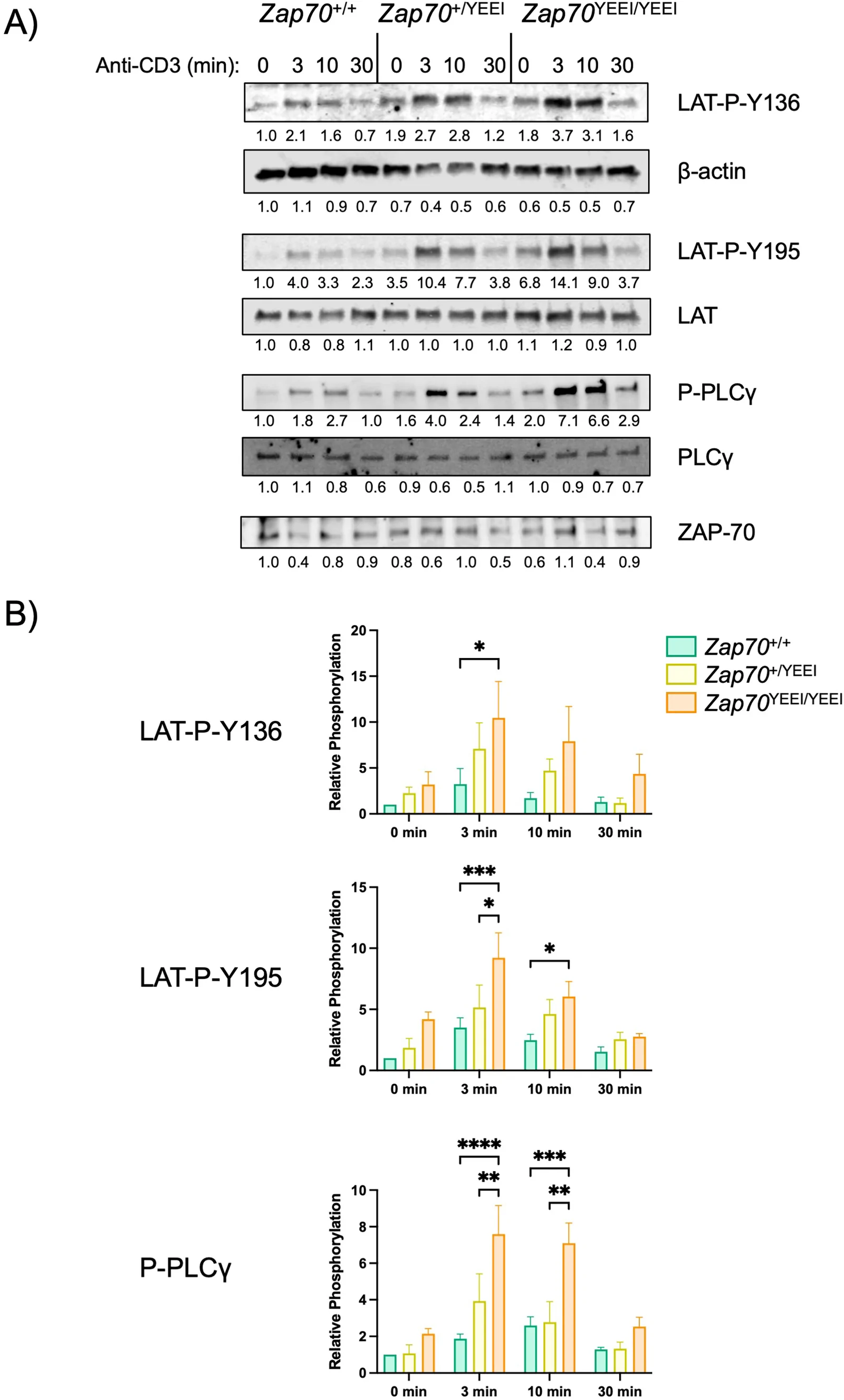
Effects of *Zap70*^YEEI^ mutation on TCR-dependent intracellular signaling of thymocytes. **(A)** 5 x 10^6^ fresh thymocytes were obtained from wild-type (*Zap70*^+/+^), heterozygous (*Zap70*^+/YEEI^) and homozygous (*Zap70*^YEEI/YEEI^) mutant mice and incubated with 0.5 μg of biotin-conjugated anti-CD3 for 30 min and then stimulated with 10 µg/ml of streptavidin for 3 min at 37°C. Cells were then lysed and analyzed for the phosphorylation of LAT or PLCγ with the indicated specific antibodies. Membranes were stripped and reanalyzed with antibodies specific for β-actin, total LAT, total PLCγ, or total ZAP-70 to show total protein load. The numbers below the images represent the relative densitometric quantification of each band, in which a relative value of 1.00 was assigned to the band corresponding to 0 min of stimulation time in cells from wild-type mice. **(B)** Diagrams representing the mean fold increase in phosphorylation in 4 independent experiments. Phosphorylation levels were normalized to total protein expression. Error bars represent the standard error of the mean. Asterisks represent statistical significance (*p < 0.05; **p<0.01; ***p<0.001; ****p<0.0001).

We further assessed the impact of the *Zap70^YEEI^* mutation on the downstream effector PLC-γ1. As anticipated, CD3 cross-linking triggered rapid PLC-γ1 phosphorylation in all genotypes, yet the intensity was markedly higher in mutant mice (**Figure 3A**). Densitometric quantification of four independent experiments confirmed that these differences were statistically significant between *Zap70*^+/+^ and *Zap70^YEEI/^*^YEEI^ mice at both the 3-minute and 10-minute time points. Similar to the LAT phosphorylation profile, *Zap70^+/^*^YEEI^ thymocytes displayed PLC-γ phosphorylation intensity that transitioned incrementally between wild-type and homozygous mutant levels. Collectively, these data show that the *Zap70^YEEI^* mutation results in a rheostat-like, dose-dependent enhancement of proximal TCR signaling components.

### Impact of *Zap70*^YEEI^ on positive selection and lineage commitment

We next assessed the impact of the *Zap70*^YEEI^ mutation on positive selection and lineage commitment. The frequency of TCRβ^high^CD5^+^ cells remained comparable across wild-type, heterozygous, and homozygous mutant mice (**Supplementary Figure 6A**). However, we observed a statistically significant increase in the overall percentage of TCRβ^high^ thymocytes in homozygous mutant mice compared to wild-type controls, accompanied by a strong trend toward higher mean channel intensity of TCRβ expression (*p* = 0.0824) (**Supplementary Figure 6B**). To investigate the developmental impact of the *Zap70*^YEEI^ mutation, we further analyzed thymic maturation based on TCRβ and CD69 expression (28, 29). By analyzing TCRβ and CD69 expression, five developmental stages (denoted I to V) can be established, and this progression has been previously validated by the gradual decline of *Rag2-GFP* activity (30). After TCR recognition of self-pMHC complexes by DP thymocytes positive selection occurs, leading to the upregulation of TCRαβ and CD69 at stages II and III (31, 32). Across the different stages (I to V) defined by TCRβ and CD69 expression, CD4 and CD8 expression patterns changed progressively, with most DP cells found in stage I and a large majority of CD4SP or CD8SP thymocytes observed in stage V (**Supplementary Figure 7A**). Our analysis of TCRβ and CD69 revealed no differences in the percentages or cell numbers at any of the above stages between wild-type and mutant mice (**Figure 4A**; **Supplementary Figures 6C, D**).

**Figure 4:**
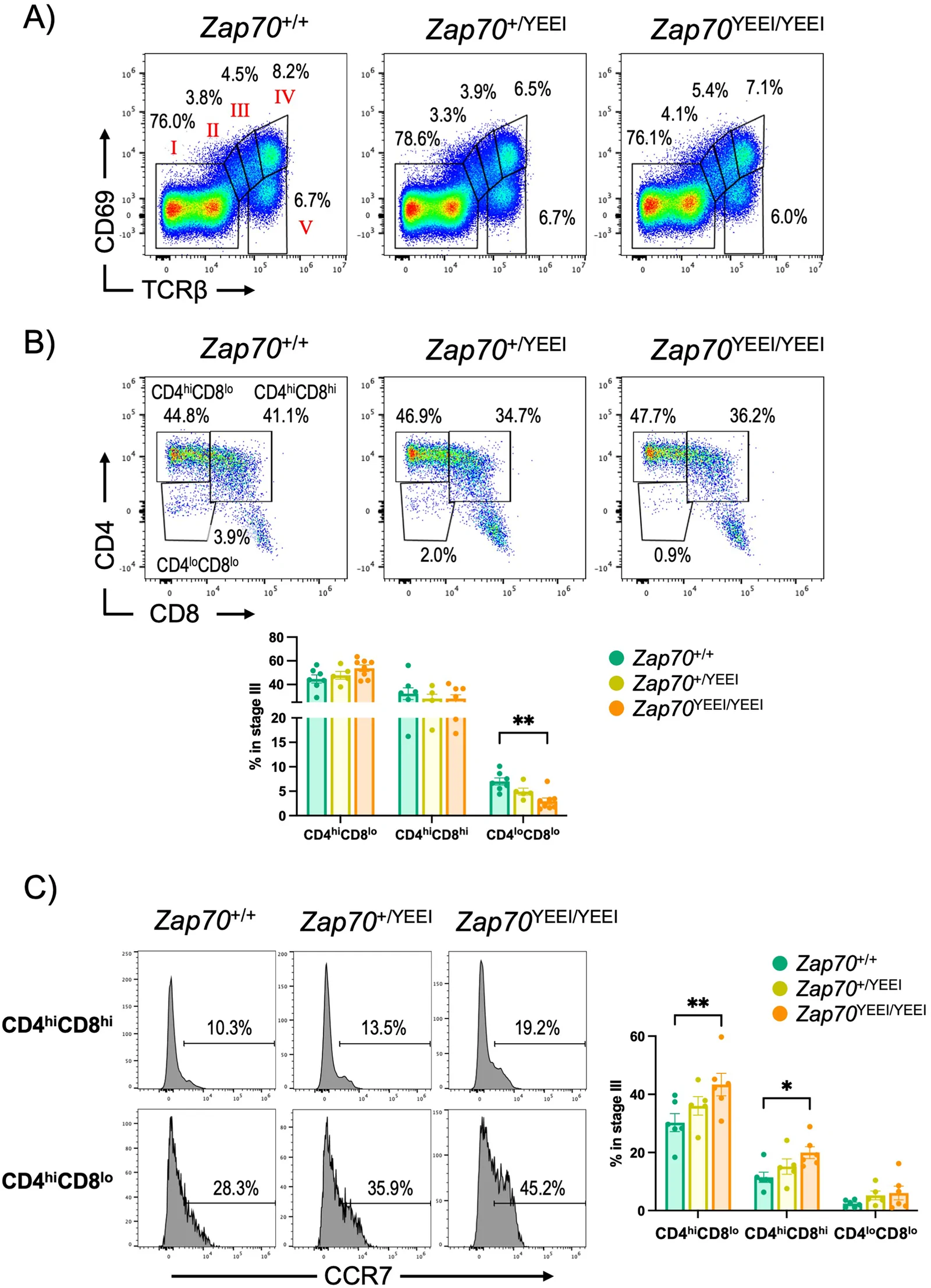
The *Zap70*^YEEI^ mutation does not alter thymic positive selection. **(A)** Thymocyte developmental stages defined by TCRβ versus CD69 expression in adult wild-type (*Zap70*^+/+^), heterozygous (*Zap70*^+/YEEI^), and homozygous (*Zap70*^YEEI/YEEI^) mice. **(B)** Flow cytometric analysis of CD4 and CD8 expression profiles in stage III cells (upper panels). The lower bar graph shows the mean percentage of CD4^hi^CD8^lo^, CD4^hi^CD8^hi^, and CD4^lo^CD8^lo^ stage III thymocytes in wild-type (n = 7), heterozygous (n = 5), and homozygous (n = 8) mice aged 6–16 weeks. Error bars represent the standard error of the mean. **(C)** CCR7 expression in subpopulations of stage III thymocytes (left panels). The bar graph on the right shows the mean percentages of CCR7^+^ cells in each of the indicated subpopulations of stage III thymocytes from wild-type (n = 6), heterozygous (n = 5), and homozygous (n =6) mutant mice aged 6–16 weeks. Brackets on each bar represent the standard error of the mean. Asterisks indicate statistical significance (*p < 0.05; **p<0.01).

Stage III is of particular functional relevance, as it represents the window for CD4/CD8 lineage commitment. According to the kinetic signaling model for CD4/CD8-lineage choice (33), positively selecting TCR signals induce CD4^hi^CD8^hi^ DP thymocytes to end *Cd8* gene expression and to convert into CD4^hi^CD8^lo^ cells. It is in these last cells that the CD4 versus CD8 lineage choice is made: if TCR signaling continues, interleukin-7 (IL-7)-mediated signaling is blocked, which induces CD4 T cell differentiation. Conversely, if TCR signaling is disrupted after CD8 downregulation in CD4^hi^CD8^lo^ cells, IL-7-mediated signaling induces co-receptor reversal, and the final differentiation into CD8^+^ T cells. We found that the percentages and absolute numbers of Stage III CD4^hi^CD8^hi^ and CD4^hi^CD8^lo^ populations were indistinguishable between heterozygous and homozygous *Zap70*^YEEI^ mice and WT mice (**Figure 4B**; **Supplementary Figure 6E**), suggesting that *Zap70*^YEEI^-mediated signaling enhancement does not detectably alter the CD4-CD8 lineage commitment program. Interestingly, we did observe a significant reduction in the CD4^lo^CD8^lo^ subpopulation in *Zap70*^YEEI/YEEI^ mice compared to wild-type mice (**Figure 4B**; **Supplementary Figure 6E**). The percentage and number of these cells in heterozygous mice had values intermediate between those of wild-type mice and homozygous mutant mice, although the difference in this case did not reach statistical significance compared to wild-type mice.

CD4^lo^CD8^lo^PD-1^+^ cells constitute an unconventional lineage that serves as a precursor to αβ-TCR^+^ DN thymocytes, which ultimately differentiate into CD8αα IELs (34). Consistent with this, PD-1 expression analysis confirmed that the majority of CD4^lo^CD8^lo^ cells were PD-1^+^, and this specific subset was significantly diminished in homozygous mutants (**Supplementary Figure 7B**). Finally, we assessed the expression of CCR7, a chemokine receptor essential for cortex-to-medulla migration. Despite the lack of gross changes in overall positive selection, the *Zap70*^YEEI^ mutation induced a significant increase in the frequency of CCR7^+^ cells within the Stage III CD4^hi^CD8^hi^ and CD4^hi^CD8^lo^ subsets (**Figure 4C**). Collectively, these findings indicate that while the *Zap70*^YEEI^ mutation enhances TCR surface expression during positive selection, it does not alter overall selection throughput or CD4/CD8 lineage choice but specifically modulates the CD4^lo^CD8^lo^ unconventional lineage.

### Characterization of medullary CD4 single-positive thymocyte maturation in *Zap70*^YEEI^ mutant mice

Given the observed upregulation of CCR7 during the CD4/CD8 lineage commitment phase in *Zap70*^YEEI^ mutant thymocytes, we next investigated whether this signaling enhancement impacted the subsequent maturation of CD4SP cells in the thymic medulla. To this end, we analyzed the expression of MHC class I (MHCI) and CD69 in CCR7^+^ CD4SP thymocytes from adult *Zap70*^+/+^, *Zap70*^+/YEEI^, and *Zap70*^YEEI/YEEI^ mice. As CD4SP thymocytes mature in the thymic medulla, they progress from a CD69^+^MHCI^−^ semi-mature (SM) stage to a CD69^+^MHCI^+^ mature 1 (M1) stage, and finally to CD69^−^MHCI^+^ mature 2 (M2) cells, which are considered fully mature and ready for emigration (35). Our analysis of CD69 and MHCI molecules in medullary CD4SP thymocytes revealed a decrease in the SM stage in homozygous mutant mice compared to wild-type animals (**Figure 5A**). The M2 population was also somewhat increased in homozygous *Zap70*^YEEI/YEEI^ mice as compared to wild-type mice (35.5% and 32.9%, respectively), although this difference did not reach statistical significance. When we analyzed the expression of active caspase 3 in CD4SP subpopulations we again observed that the *Zap70*^YEEI^ mutation induced a decrease in the percentage of apoptotic cells between wild-type and mutant mice, with statistically significant differences in M1 and M2 populations (**Figure 5B**; **Supplementary Figure 8B**). Simultaneous analysis of CD25 expression revealed a strong trend toward an increased percentage of CD25^+^ cells in the M2 subpopulation of homozygous *Zap70*^YEEI/YEEI^ mice compared to wild-type controls (*p* = 0.084; **Supplementary Figures 8A, B**). Collectively, these findings demonstrate that the *Zap70*^YEEI^ mutation results in enhanced survival of medullary CD4SP cells and suggests a bias toward CD25^+^ expansion within the fully mature (M2) CD4SP thymocyte subset, consistent with an increased pool of thymic Treg precursors in homozygous mutant mice.

**Figure 5:**
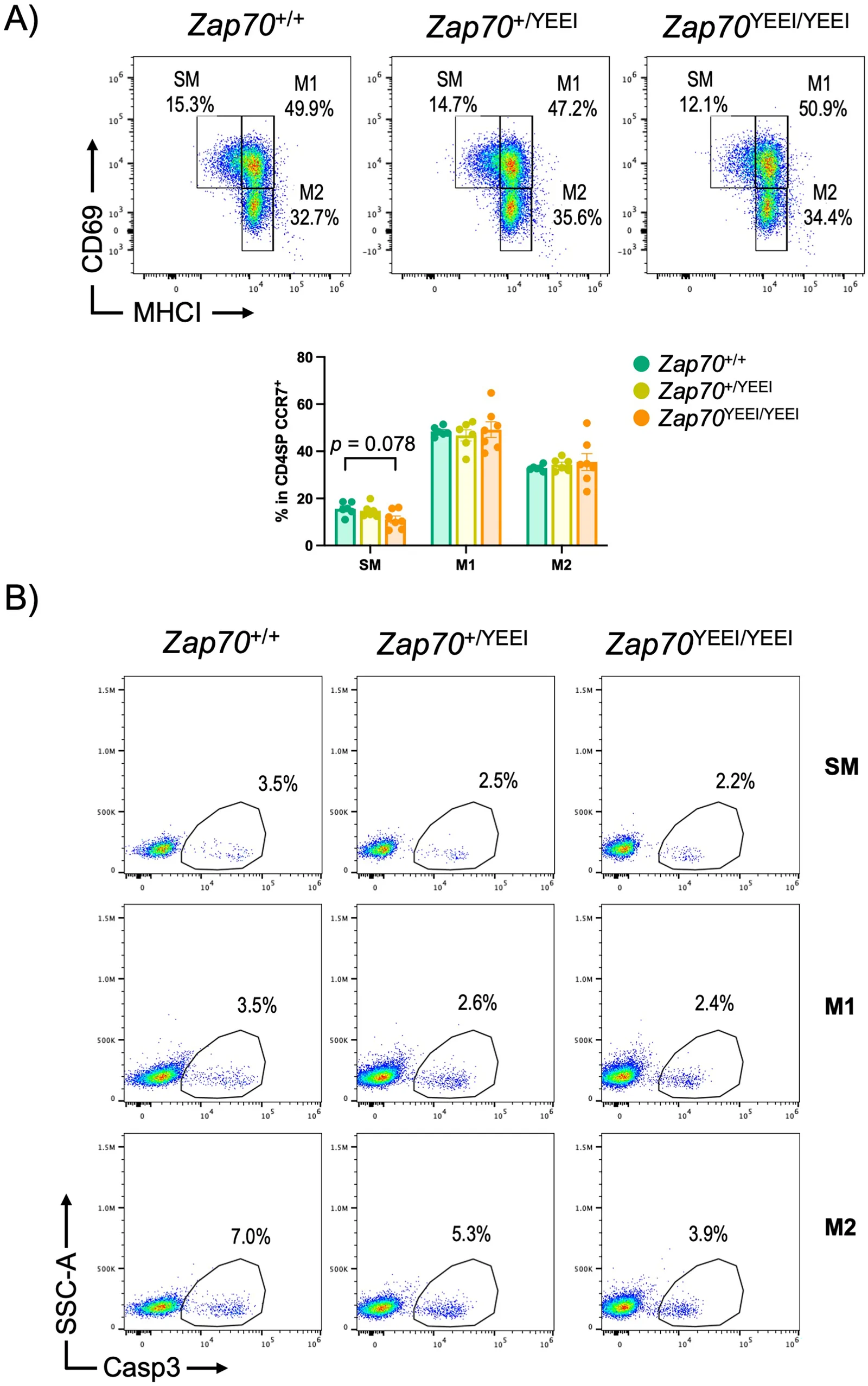
Effect of the *Zap*^YEEI^ mutation on the last steps of medullary CD4 SP thymocyte development. **(A)** Analysis of the expression of CD69 and MHC class I (H-2K^b^) by medullary (CCR7^+^) CD4 single positive thymocytes in adult wild-type (*Zap*^+/+^), heterozygous (*Zap*^+/YEEI^), and homozygous (*Zap*^YEEI/YEEI^) mutant mice (upper dot plots). The lower bar graph represents the mean percentage of semi-mature (SM), mature 1 (M1) and mature 2 (M2) cells in wild-type (n = 6), heterozygous (n = 6), and homozygous (n = 7) mice aged 6–16 weeks. Error bars represent the standard error of the mean. **(B)** Dot plots showing active caspase 3 expression in SM, M1 and M2 cells from adult wild-type, heterozygous, and homozygous mutant mice. One representative experiment is shown.

### Analysis of peripheral T cell populations in *Zap70*^YEEI^ mice

Having characterized the role of the *Zap70*^YEEI^ mutation on intrathymic T cell development we next examined whether the *Zap70*^YEEI^ mutation influenced the composition and phenotype of T cells in the peripheral lymphoid organs. Analysis of the splenic compartment revealed a trend toward increased total T cell cellularity in homozygous *Zap70*^YEEI/YEEI^ mice compared to wild-type (*Zap70*^+/+^) controls, though this difference did not reach statistical significance in 6–16 weeks old mice (*p* = 0.1680; **Supplementary Figure 2C**). In contrast, splenocyte numbers in heterozygous Zap70^+/YEEI^ mice were significantly lower than those in homozygous mutants (**Supplementary Figure 2C**). Regarding the distribution of TCRβ^+^ and TCRγδ^+^ lineages, the *Zap70*^YEEI^ mutation did not alter the frequency of TCRβ^+^ cells. However, the percentage of TCRγδ^+^ cells was significantly elevated in the spleens of homozygous mutants, a phenotype absent in heterozygous mice (**Figure 6A**). Consistent results were obtained from lymph node analysis (**Supplementary Figure 9A**). Notably, TCRγδ frequencies in wild-type and heterozygous *Zap70*^+/YEEI^ mice were strikingly similar whereas homozygous mutants uniquely showed significantly higher TCRγδ percentages. Despite this expansion, the phenotype of γδ T cells remained consistent across all the three genotypes, with the majority being CD4^−^CD8^−^ and CD27^+^ (**Supplementary Figures 9B, C**). Furthermore, and in contrast to our previous observations in LAT-knockout mice (36), the intensity of CD27 expression was comparable across all genotypes (**Supplementary Figure 9C**).

**Figure 6:**
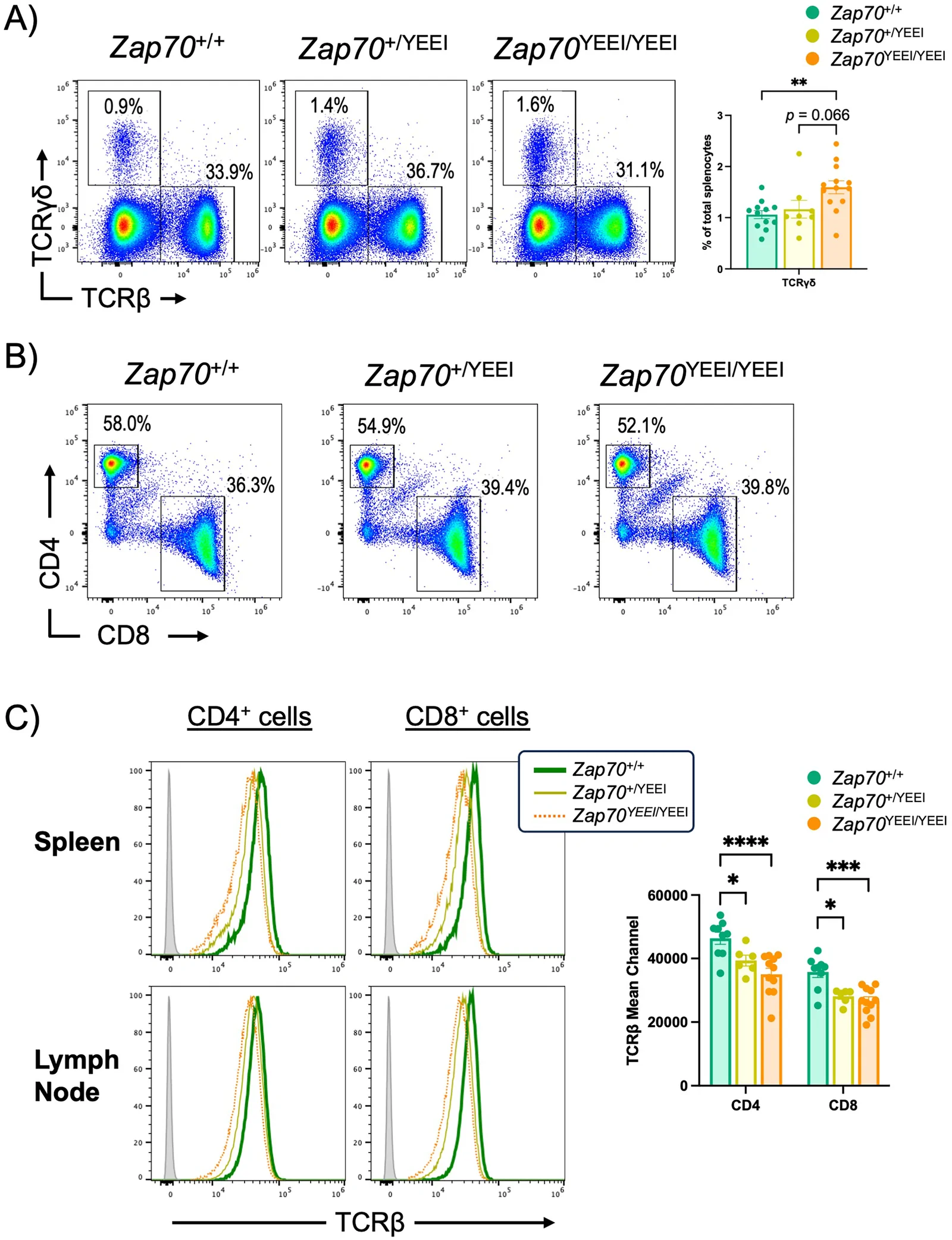
Analysis of splenocytes of *Zap70*^YEEI^ mutant mice. **(A)** Viable splenocytes from adult wild-type (*Zap70*^+/+^), heterozygous (*Zap70*^+/YEEI^), and homozygous (*Zap70*^YEEI/YEEI^) mutant mice were analyzed for TCRβ and TCRγδ expression. The bar graph on the right shows the mean percentage of TCRγδ^+^ thymocytes in the spleens from wild-type (n = 12), heterozygous (n = 8), and homozygous (n = 13) mice aged from 6–16 weeks. Error bars represent the standard error of the mean. **(B)** TCRβ^+^ splenocytes from wild-type, heterozygous, and homozygous mice were analyzed by flow cytometry for the expression of CD4 and CD8. One representative experiment is shown. **(C)** TCRβ expression intensity analyzed by flow cytometry in CD4^+^ and CD8^+^ lymphocytes from the spleens or lymph nodes of wild type (green thick line), heterozygous (yellow thin line), and homozygous (dotted orange line) mutant mice (histograms on the left). The grey shadowed line represents the isotype control. The bar graph on the right shows the average values of the mean channel for TCRβ expression in CD4^+^ and CD8^+^ splenocytes from wild-type (n = 9), heterozygous (n = 6), and homozygous (n = 11) mutant mice. Error bars represent the standard error of the mean. Asterisks indicate statistical significance (*p < 0.05; **p< 0.01; ***p< 0.001; ****p< 0.0001).

In the αβ T cell lineage, the distribution remained largely stable, with no significant differences in the relative percentages of CD4^+^ or CD8^+^ T cells in the spleen or lymph nodes across the three genotypes (**Figure 6B**; **Supplementary Figure 9D**). However, the *Zap70*^YEEI^ mutation significantly impacted surface receptor density. We detected a consistent reduction of approximately 20% in the mean fluorescence intensity (MFI) of TCRβ on both CD4^+^ and CD8^+^ T cells from the spleen or lymph nodes (**Figure 6C** and **Supplementary Figure 10A**). This downregulation is consistent with prior reports in CD8-driven, gain-of-function *Zap70*^YEEI^ transgenic mice (17). Interestingly, this reduction in TCR intensity was lineage specific, as it was not observed in γδ T cells from either the spleen or lymph nodes (**Supplementary Figure 10B**).

In addition to maintaining normal TCR levels, the expanded γδ T-cell population in *Zap70*^YEEI/YEEI^ lacked an activated phenotype, remaining negative for both CD69 and CD25 (**Supplementary Figure 10C**). Therefore, these data indicate that while the *Zap70*^YEEI^ mutation drives a specific expansion of the peripheral γδ T-cell compartment and a downmodulation of αβ-TCR surface levels, it does not induce the spontaneous activation or lymphoproliferative disorders associated with other TCR-signaling mutants.

### Phenotypic characterization of peripheral Treg cells in *Zap70*^YEEI/YEEI^ mice

Considering the increased thymic TCRβ^+^CD4^+^CD25^+^ frequencies found in *Zap70*^YEEI/YEEI^ mice, we examined whether this phenotype extended to the peripheral Treg cell compartment. Flow cytometric analysis of the spleen revealed that the percentage of TCRβ^+^CD4^+^CD25^+^ cells was significantly higher in homozygous mutants than in wild-type controls (**Figure 7A**). FoxP3 expression confirmed such a robust expansion of the Treg population in *Zap70*^YEEI/YEEI^ mice (**Figure 7B**). Quantitatively, the total number of splenic Treg cells in homozygous mutants was approximately twice that of wild-type littermates (1.68 ± 0.26 x 10^6^ vs. 0.85± 0.17 x 10^6^, respectively; **Supplementary Figure 11A**). A comparable expansion was also observed in the lymph nodes of *Zap70*^YEEI/YEEI^ mice (data not shown). To further characterize this expanded Treg cell pool, we subdivided the Treg cells into distinct subsets based on their maturation and activation status: central Tregs (cTregs), characterized as Ly6C^−^CD62L^hi^ or Ly6C^+^CD62L^hi^ cells, and effector Tregs (eTregs), defined as Ly6C^−^CD62L^lo^ (37, 38). Our analysis revealed that eTreg population was significantly enriched in absolute cell numbers in the spleens of *Zap70*^YEEI/YEEI^ mice, whereas its frequency remained comparable to controls (**Figure 7C**; **Supplementary Figure 11B**). Notably, as Ly6C expression on peripheral Treg cell cells is negatively regulated by tonic TCR signaling (37), the altered Ly6C profile observed here may be a direct consequence of the *Zap70*^YEEI^ mutation. When evaluating absolute cell numbers across all subsets, *Zap70*^YEEI/YEEI^ mice exhibited significant increases not only in eTreg cells but also in both Ly6C^+^ and Ly6C^−^ cTreg populations (**Supplementary Figure 11B**). This systematic increase across all subsets appears to be driven primarily by the overall expansion of the total Treg pool in homozygous mutants. Finally, we assessed the expression of CD69 and KLRG1, markers associated with high suppressive effector Tregs. While the frequencies of CD69^+^ and KLRG1^+^ cells within the Treg compartment were comparable across all genotypes (**Figure 7D**; **Supplementary Figures 11C, D**, upper panel), the absolute number of CD69^+^ eTregs was significantly elevated in *Zap70*^YEEI/YEEI^ mice relative to wild-type controls. Taken together, these data demonstrate that the *Zap70*^YEEI^ mutation drives a broad numerical expansion of the peripheral Treg compartment without modifying their expression of characteristic suppressive effector markers.

**Figure 7:**
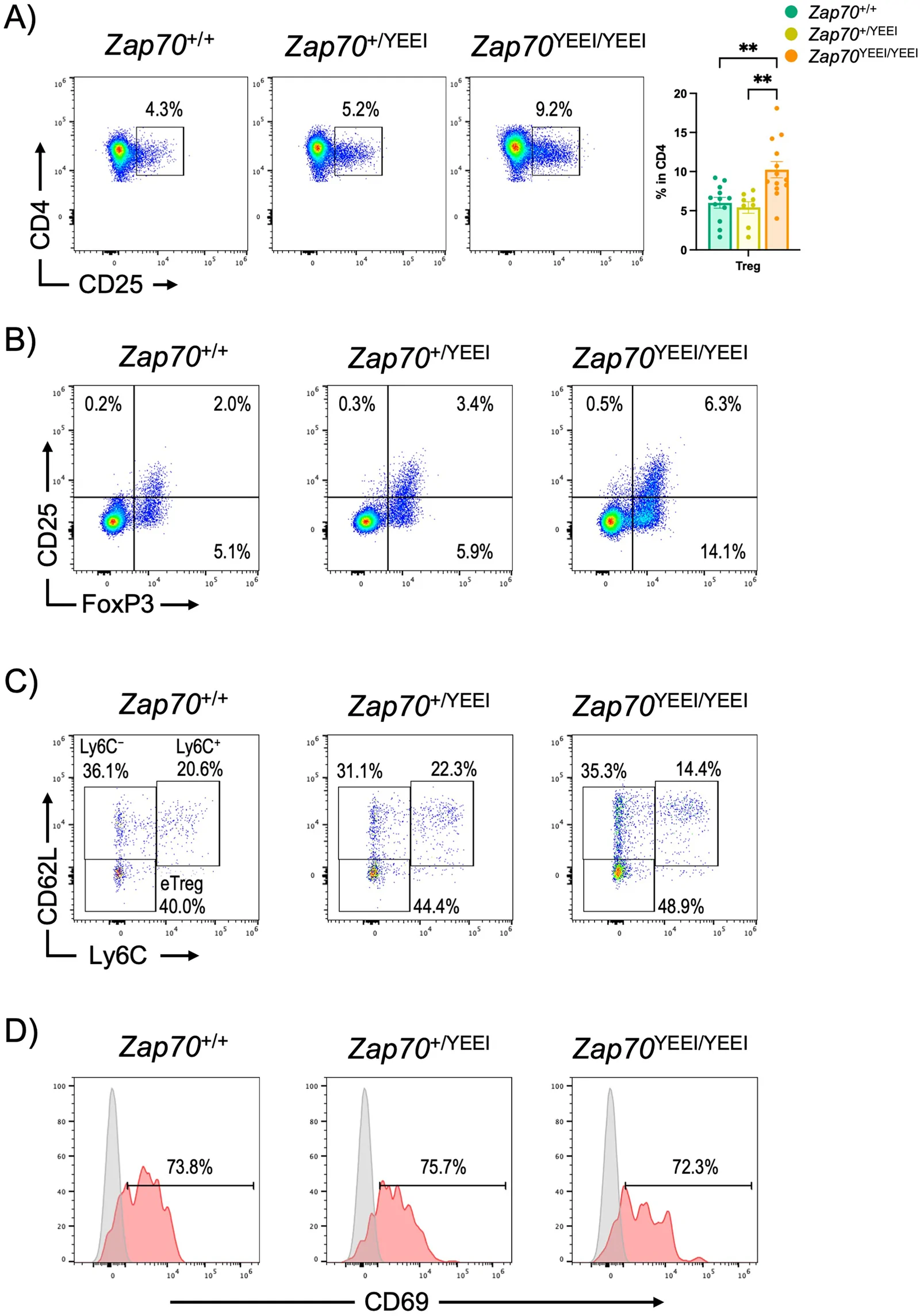
Augmentation and phenotypic remodeling of splenic Treg cells in *Zap*^YEEI/YEEI^ mutant mice. **(A)** The Treg compartment was analyzed within the TCRβ^+^CD4^+^ population based on CD25 expression in spleens from wild-type (*Zap70*^+/+^), heterozygous (*Zap70*^+/YEEI^), and homozygous (*Zap70*^YEEI/YEEI^) mice. The bar graph (right) shows the mean frequency of TCRβ^+^CD4^+^CD25^+^ splenocytes in wild-type (n = 12), heterozygous (n = 8), and homozygous (n= 13) mutant mice, aged 6–16 weeks. Error bars indicate SEM. Asterisks indicate statistical significance (** *p*<0.01). **(B)** Representative dot plots of FoxP3 and CD25 expression within TCRβ^+^CD4^+^ splenic lymphocytes from the indicated genotypes. **(C)** Analysis of Treg cell subpopulations defined by Ly6C and CD62L expression within TCRβ^+^CD4^+^CD25^+^ cells from the spleens of *Zap70*^+/+^, *Zap70*^+/YEEI^, and *Zap70*^YEEI/YEEI^ mice. One representative experiment is shown. **(D)** Representative histograms of CD69 expression in effector Treg (eTreg; Ly6C^−^CD62L^−^) cells from spleens of the indicated genotypes.

### Phenotypic analysis of conventional T lymphocyte subsets

We next investigated the impact of the *Zap70*^YEEI^ mutation on the conventional T cell compartment, focusing on the transition from naive to memory phenotypes. Analysis of memory markers within the splenic TCRβ^+^CD4^+^CD25^−^ T cell population revealed a significant shift toward a CD44^hi^CD62L^−^ memory phenotype in homozygous *Zap70*^YEEI/YEEI^ mice. Specifically, we observed a marked increase in the percentage of these memory CD4^+^ cells (mCD4) in homozygous mutants compared to wild-type controls (**Figure 8A**). In contrast, heterozygous mutants maintained a distribution of naive and memory populations comparable to wild-type animals. Comprehensive analysis of mice aged from 6 to 16 weeks (n = 10 wild-type, n = 7 heterozygous, and n = 10 homozygous mice) confirmed that both the frequency of memory CD4^+^ T cells were significantly elevated in homozygous mutants, which was accompanied by a strong trend toward higher absolute cell numbers (*p* = 0.054, **Figure 8B**), underscoring a lineage-specific effect of the mutation on CD4^+^ T cell homeostasis. In contrast, the splenic CD8^+^ T cell compartment appeared relatively less affected by the *Zap70*^YEEI^ mutation. There were no detectable differences in the percentage of central memory CD8^+^ cells (CD44^hi^CD62L^+^) across any of the three genotypes (**Figures 8C, D**). Interestingly, whereas the relative proportions of naive CD8^+^ splenocytes (CD44^lo^CD62L^+^) remained stable, their absolute number was elevated in homozygous *Zap70*^YEEI/YEEI^ mice as compared to wild-type controls. Given that the percentages were unchanged, this numerical increase likely reflects the higher overall cellularity observed in the spleens of these mutant mice rather than a targeted expansion of the CD8^+^ naive pool. Together, these results suggest that while the *Zap70*^YEEI^ mutation skews the CD4^+^ lineage toward a memory phenotype, its impact on the CD8^+^ compartment is primarily quantitative, a feature corresponding to a global increase in the overall peripheral lymphoid pool.

**Figure 8:**
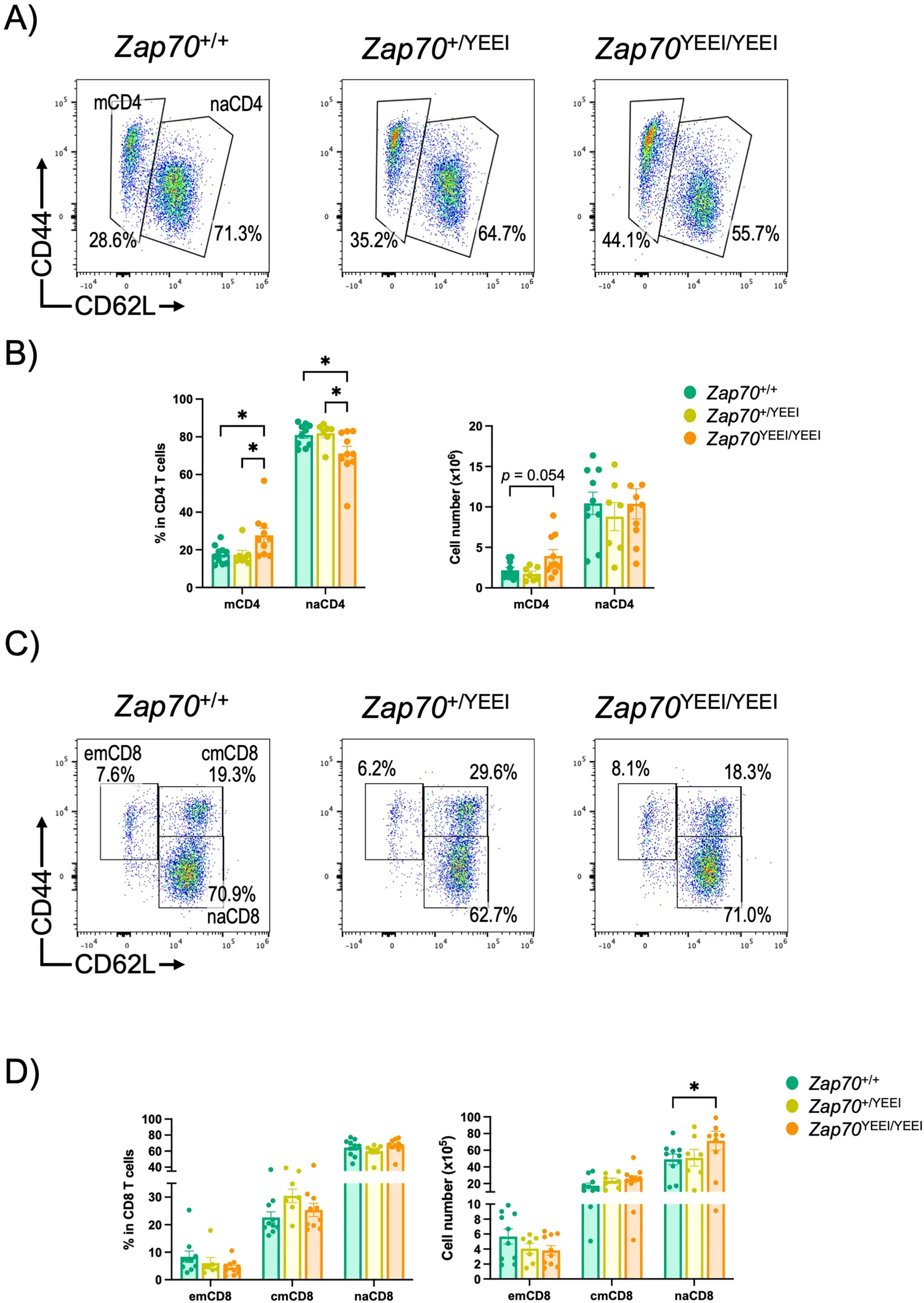
Analysis of memory and naive T cell populations in mutant mice. **(A)** Representative flow cytometry plots of splenic T cells gated on CD44 and CD62L. Gates define memory (mCD4) and naive (naCD4) populations. Numbers indicate the percentage of cells within each gate. **(B)** Quantification of T cell subsets. Left: Frequency of mCD4 and naCD4 as a percentage of total splenic CD4^+^ T cells. Right: Absolute cell numbers of mCD4 and naCD4 populations. 10 wild-type mice, 7 heterozygous mice, and 10 homozygous mice aged 6–16 weeks were analyzed. Bars indicate SEM. **(C)** Representative flow cytometry plots of splenic CD8^+^ T cells gated on CD44 and CD62L. Gates define effector memory (emCD8), central memory (cmCD8), and naive (naCD8) populations. **(D)** Quantification of T cell subsets. Left: Frequency of emCD8, cmCD8, and naCD8 as a percentage of total splenic CD8^+^ T cells. Right: Absolute cell numbers of each subset. Data points represent individual mice; bars indicate SEM. Asterisks indicate statistical significance (*p < 0.05).

### Functional assessment of peripheral T cell activation and proliferation

To determine whether the *Zap70*^YEEI^ mutation alters the functional responsiveness of mature T lymphocytes, we assessed their activation and proliferative capacity *in vitro*. Purified splenic CD3^+^ T cells from wild-type, heterozygous, and homozygous mutant mice were labeled with CellTrace Violet (CTV) and stimulated with anti-CD3/CD28-coated microbeads at two different bead-to-cell ratios, following established protocols (39). To ensure high-resolution analysis of specific subsets, T cells were co-stained for CD4 and CD8, while Annexin V staining was used to exclude apoptotic cells from the analysis. Early T cell activation was evaluated by monitoring the surface expression of CD69 after 24 hours of stimulation. As shown in **Figure 9A**, both CD4^+^ and CD8^+^ T cells from all three genotypes (*Zap70*^+/+^, *Zap70^+/^*^YEEI^, and *Zap70*^YEEI/YEEI^) exhibited comparable levels of CD69 induction. Consistent with these activation profiles, the proliferative capacity of both CD4^+^ and CD8^+^ T cells remained unaffected by the mutation, with no significant differences in CTV dilution patterns observed across the three genotypes (**Figure 9B**). These findings indicate that while the *Zap70*^YEEI^ mutation influences peripheral T cell homeostasis, it does not modify the ability of mature T cells to be activated or to proliferate in response to TCR-mediated stimulation.

**Figure 9:**
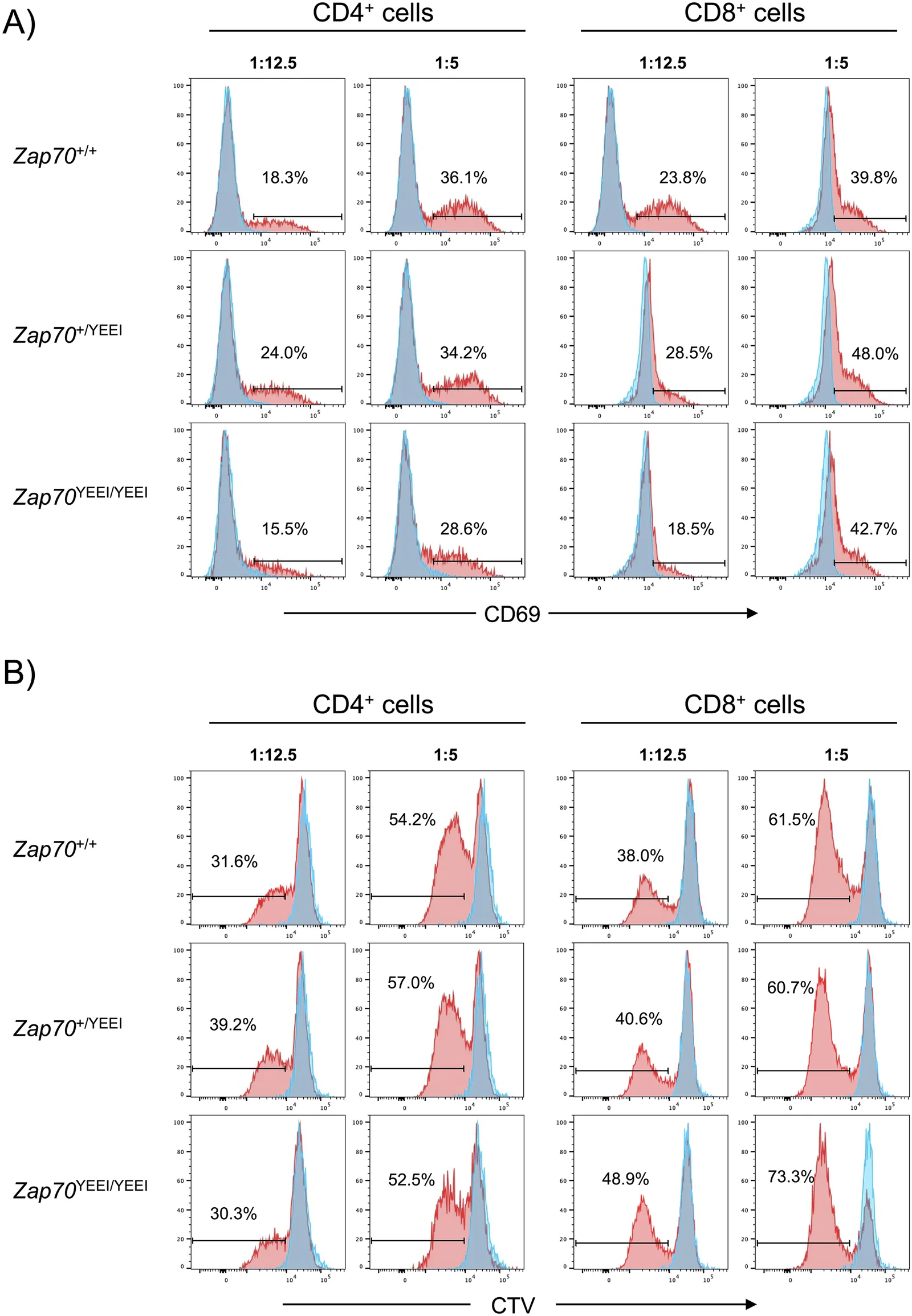
Ex vivo stimulation assays. **(A)** T cells purified from the spleens of wild-type (*Zap70*^+/+^), heterozygous (*Zap70*^+/YEEI^), and homozygous (*Zap70*^YEEI/YEEI^) mutant mice were stimulated with the indicated bead:cell ratios of anti-CD3/CD28 microbeads, and analysis of CD69 surface expression 24H post-stimulation was performed on live cells (Annexin V negative). CD69 expression in CD4^+^ and CD8^+^ cell populations are shown separately. The numbers indicated in each histogram represent the percentage of CD69 positive cells (red histograms). Blue histograms show CD69 expression in unstimulated cells. One representative experiment is shown (n=3). **(B)** Analysis of proliferation performed at 72H post-stimulation. Decrease of CTV staining indicates cell proliferation. Blue histograms correspond to unstimulated cells and red histograms to anti-CD3/CD28 stimulated cells. Proliferative capacity was analyzed separately in CD4^+^ and CD8^+^ cell populations. The numbers shown in each histogram represent the percentage of cells that have proliferated. One representative experiment is shown (n=3).

## Discussion

Thymic development constitutes a biological process by which a large repertoire of T lymphocytes is generated. At the same time, given the stochastic nature of TCR rearrangements, autoreactive T cells can be generated during their maturation. Intracellular TCR signaling determines the fate of thymocytes, such that those that recognize self-antigens with high affinity (and which could pose a threat to the body) must be eliminated or differentiate into regulatory T cells. It is therefore particularly important to analyze how the intracellular signals leading to these outcomes are regulated and controlled. ZAP-70 is a protein tyrosine kinase that plays a crucial role in T-cell activation and development, and its absence causes thymic development to arrest at the double-positive stage (8). While the loss-of-function *Zap70*^Y319F^ mutation impairs downstream signaling by disrupting the ZAP-70/LCK interaction, replacing the native Y319 sequence with a high-affinity YEEI motif (*Zap70*^YEEI^) enhances this interaction and drives gain-of-function TCR signaling (15, 16).

Remarkably, the native Y319-SDP sequence in ZAP-70 is exquisitely conserved across evolutionary time, displaying 100% amino acid identity across humans, mice, rats, dogs, cows, chimpanzees, and macaques. This strict phylogenetic conservation suggests strong evolutionary pressure to preserve a very specific signaling threshold during T cell development. By naturally maintaining an “SDP” sequence over higher-affinity alternatives such as “YEEI,” the mammalian immune system appears to have calibrated ZAP-70/LCK binding kinetics to maintain fine-tuned signaling windows, avoiding over-activation by self-antigens while preserving essential selection thresholds.

Transgenic mice expressing the *Zap70*^YEEI^ mutant revealed that the LCK-mediated phosphorylation of the ZAP-70 Y319 residue plays a dual role: it initially promotes activation by relieving autoinhibition but subsequently triggers negative feedback to diminish long-term signaling and IFN-γ production (17). This dual mechanism appears to dynamically tune T-cell sensitivity to prevent overactivation by high-affinity autoantigens. However, because transgene expression was restricted to the CD8^+^ single-positive stage onward, these findings are strictly limited to CD8^+^ T cells, leaving the role of this regulatory axis in broader thymic development unknown. Therefore, analyzing a knock-in mouse strain with this same mutation was of interest for studying the physiological effects of increased interaction between LCK and ZAP-70.

Our results demonstrate that the *Zap70*^YEEI^ mutation does not grossly impair thymic development, as evidenced by comparable percentages of double-negative (DN), double-positive (DP), and single-positive (SP) populations between mutant and wild-type mice. However, restricting our analyses to mice prior to thymic involution (6–16 weeks of age) revealed a statistically significant increase in the frequency of TCRβ^high^ thymocytes in homozygous *Zap70*^YEEI/YEEI^ mutants, accompanied by a strong trend toward higher TCRβ mean intensity levels (**Supplementary Figure 6A**). While the percentage of “signaled” (TCRβ^+^CD5^+^) thymocytes exhibited a modest elevation in mutant mice, this increase did not reach statistical significance. Nevertheless, the preferential expansion of the TCRβ^high^ population points to enhanced positive selection efficiency and altered baseline TCR signal integration *in vivo*. This lack of early phenotypic disruption during the DN stage may be attributed to the high expression of Syk during pre-TCR signaling stages, which is subsequently downregulated once the pre-TCR checkpoint is passed (40). Despite unaffected gross development, we observed a significant augmentation of regulatory T (Treg) precursors in both heterozygous and homozygous mutant mice. Interestingly, this precursor expansion translated to a higher frequency of peripheral Treg cells exclusively in homozygous mutants, suggesting a gene-dosage effect where maximum ZAP-70 gain-of-function signaling is required to stably alter peripheral Treg homeostasis.

It is particularly striking that thymic apoptosis is reduced in *Zap70*^YEEI^ mutant mice. While a decrease in death-by-neglect was expected in a gain-of-function mutant, the enhancement of ZAP-70-mediated signaling should have amplified negative selection. These findings do not fully align with a recent report (41), which demonstrated that higher ZAP-70 expression levels (whether physiologically in adult mice compared to neonates, or genetically via a transgenic (*Zap70*^AS^) model) correlate with increased negative selection and reduced Treg cell differentiation. Conversely, they showed that lower ZAP-70 levels (in neonates and *Zap70* heterozygotes) favored Treg development. Although the mutation in our model does not alter ZAP-70 expression, the phenotype of a gain-of-function mutant would theoretically be expected to mirror a high-expression model. Instead, the *Zap70*^YEEI^ mutant paradoxically exhibits increased Treg cell frequencies alongside reduced negative selection and death-by-neglect.

However, our observation that the gain-of-function mutation in *Zap70*^YEEI^ mice leads to an increase in the frequency of Treg cell precursors is consistent with previous reports. SKG and ZAC mutant mice are knock-in mice strains in which point mutations have been introduced into ZAP-70 which decrease its affinity for phosphorylated ITAMs (42, 43). SKG and ZAC mice spontaneously develop autoimmune disease because of a shift of the TCR repertoire of T conventional cells toward higher affinities for thymic self-pMHCs. Interestingly, although these strains show increased frequency of peripheral Treg cells (presumably as a secondary effect of their systemic inflammation), they show reductions in the frequency of CD4^+^FoxP3^+^ thymic populations. Consequently, if mutations that impair the functional capacity of ZAP-70 result in a decrease in Treg cell precursors, the increase we observed in *Zap70*^YEEI^ mice would be the expected outcome. However, the basis of the reduced negative selection observed in *Zap70*^YEEI^ mice remains unresolved. It has been shown that clonal deletion occurs before Treg generation (44) and that restricting ZAP-70 expression to pre-selection double-positive thymocytes abrogates Treg generation while leaving clonal deletion unaffected. These data suggest that reduced ZAP-70 expression inversely correlates with Treg differentiation, and this happens before negative selection. Gain-of-function mutants such as *Zap70*^YEEI^ do not just make TCR signaling stronger, they can change its nature. It has been shown that negative selection requires rapid, high amplitude, bursts of signaling, whereas Treg induction is driven by lower, sustained, signaling (45). According to this, *Zap70*^YEEI^ may be producing sustained signaling that does not trigger cell elimination, thereby favoring the expansion of the Treg compartment at the expense of clonal elimination. Furthermore, it remains possible that the unconstrained endogenous TCR repertoire in *Zap70*^YEEI^ mice undergoes a compensatory structural shift during selection, favoring TCRs with lower intrinsic affinity for self-pMHC to counterbalance the intracellular gain-of-function signaling.

Commitment to the γδ lineage remained unaffected in the thymi of *Zap70*^YEEI^ mutant mice, as evidenced by the similar percentages and numbers of TCRγδ^+^ thymocytes in mutant and wild-type mice. However, the percentage of TCRγδ^+^ cells was significantly elevated in the spleens of homozygous mutants, a phenotype absent in heterozygous mice. These cells lacked an activated phenotype and did not undergo the lymphoproliferation characteristic of a LAT knock-in strain in which its three carboxy-terminal tyrosines had been mutated to phenylalanine (46). Consequently, these findings demonstrate that the *Zap70*^YEEI^ mutation drives a targeted expansion of peripheral γδ T cells without leading to a lymphoproliferative disorder as seen in alternative TCR-signaling mutants.

Of interest, the *Zap70*^YEEI^ mutation induces in the periphery an increased frequency of memory CD4 T cells and a reduction of approximately 20% in the MFI of TCRβ on both CD4^+^ and CD8^+^ T cells. The lineage-specific expansion observed in our model may be explained by the differential sensitivity of T-cell subsets to persistent TCR stimulation. Mature CD8^+^ T cells are characterized by a significantly higher baseline susceptibility to activation-induced cell death (AICD) than CD4^+^ T cells (47). While hyper-reactive signaling via the *Zap70*^YEEI^ mutation allows CD4^+^ cells to accumulate as memory pools, chronic engagement in CD8^+^ T cells readily triggers destructive apoptotic mechanisms, such as TNF/TNFR2-mediated pathways (48) and the severe downregulation of anti-apoptotic factors like c-FLIP(S) (49). Consequently, defining how these distinct subsets balance activation against apoptosis under chronic signaling conditions will provide critical insights into T-cell homeostasis and the design of fine-tuned immunotherapies.

With regard to the functional analysis of peripheral T lymphocytes, our data demonstrate that both proliferative capacity and the induction of CD69 expression were comparable between wild-type (WT) and *Zap70*^YEEI^ mutant mice. Given that the *Zap70*^YEEI^ mutation enhances TCR signaling and augments the frequency of CD4^+^ memory cells, these equivalent functional responses were unexpected. While gain-of-function mutations in other key signaling nodes, such as the LAT adaptor (*Lat*^G135D^ knock-in mice), have been shown to favor heightened T-cell responses (50, 51), several mechanisms may account for the unexpected phenotypic discrepancy observed in our *Zap70*^YEEI^ model. First, this discrepancy may stem from the reduced surface TCR expression observed on both CD4^+^ and CD8^+^ T cells from heterozygous and homozygous mutant mice. Second, the homeostatic response may be constrained by an augmented peripheral regulatory Treg cell population. Supporting this hypothesis, we observed that the effector Treg (eTreg) subpopulation was significantly enriched in the spleens of *Zap70*^YEEI/YEEI^ mice in both frequency and absolute cell number. Because Ly6C expression on peripheral Treg cells is negatively regulated by tonic TCR signaling (37), the altered Ly6C profile identified in this study may represent a direct downstream consequence of the hyperactive *Zap70*^YEEI^ mutation, potentially enhancing Treg-mediated suppression and mitigating peripheral hyper-reactivity.

In conclusion, our characterization of the *Zap70*^YEEI^ knock-in mouse model establishes that enhancing the structural interaction between LCK and ZAP-70 fundamentally skews T-cell selection and peripheral homeostasis without compromising gross thymic architecture. While bulk biochemical analyses like Western blotting effectively confirm the unambiguous molecular identity of downstream signaling nodes across primary populations, exploring the single-cell phosphorylation kinetics across sorted subsets will further refine our understanding of subset-specific alterations. Ultimately, rather than universally amplifying downstream responses, this specific gain-of-function mutation alters the underlying dynamics of TCR signaling, potentially driving sustained, lower-amplitude signals that selectively expand the thymic regulatory T (Treg) cell precursor pool at the expense of clonal deletion. In the periphery, this hyperactive signaling baseline is countered by robust homeostatic adaptations, including the downregulation of surface TCR expression and a significant enrichment of suppressing effector Treg populations. This dual architecture successfully limits overt systemic inflammation and preserves baseline lymphocyte proliferative capacities. Ultimately, these findings underscore that engineering the ZAP-70-LCK signaling axis does not merely amplify signal magnitude but alters the nature of the signal itself, offering critical insights into how fine-tuned intracellular kinetics dictate the delicate balance between immunological tolerance and peripheral reactivity.

## Methods

### Antibodies and reagents

The anti-CD3, -CD4, -CD5, -CD8, -CD11b, -CD24, -CD25, -CD27, -CD44, -CD62L, -CD69, -CD73, -CD161c (NK1.1), -CCR7, FoxP3, -H-2Kb/H2-Db, -I-A/I-E mouse MHC class II, -KLRG1, -Ly-6C, -TCRβ, and -TCRγδ fluorochrome-coupled antibodies used for cytometry were from BioLegend (San Diego, CA, USA). The FITC Active Caspase-3 Apotosis Kit was from BD Pharmingen (San Diego, CA, USA). Antibodies specific for P-PLC-γ1 (Y783), P-Y191-LAT, total LAT, total ZAP-70, and β-Actin were from Cell Signaling Technologies (Danvers, MA, USA). Anti-P-Y132-LAT antibody was from Abcam (Cambridge, MA, USA). PLC-γ1 antibody was from Santa Cruz Biotechnology (Heidelberg, Germany). CellTraceTM Violet and Dynabeads^®^ Mouse T-Activator CD3/CD28 reagents were from Invitrogen (ThermoFisher Scientific, Waltham, MA, USA). Biotin anti-mouse CD3ϵ and streptavidin were from BioLegend.

### Animals

Mice were maintained and used at the local Animal Supply Services (SEPA, University of Cadiz). Animal care and handling followed the guidelines of the European Union Council (2010/63/EU, 86/609/UE) on the use of laboratory animals. Experimental procedures were approved by the local Animal Care and Ethics Committee (University of Cadiz, Cadiz, Spain) and the Ministry of Agriculture, Fisheries and Rural Development (Junta de Andalucía, Spain). To prevent confounding effects associated with age-dependent thymic involution, all experimental procedures and quantitative analyses were strictly conducted using mice between 6 and 16 weeks of age.

### *Zap70*^YEEI^ mutation

Mutagenesis was performed on a 220-bp SmaI-SphI fragment containing the relevant segment of exon 8. By replacing the sequence coding for the YSDP peptide with one encoding YEEI, a new Sau3AI restriction site was introduced in exon 8, facilitating the subsequent identification of *Zap70*^YEEI^ mutant mice. This modification did not alter the ZAP-70 amino acid sequence outside of the YEEI mutation. A loxP-flanked NeoR cassette was introduced into the intron 5′ of exon 3. The final targeting construct contained 1.6 kb and 5 kb of homologous sequences 5′ and 3′ of the *NeoR* gene, respectively (**Supplementary Figure 1**). Following electroporation of E14 129/Ola ES cells and G418 selection, recombinant colonies were identified via Southern blot using probes identical to those described for screening the ZAP-70-Y315F knock-in strain (13). For the *Zap70*^YEEI^ mutation, the 3′ probe hybridized to a 7.3-kb wild-type fragment or an 8.8-kb recombinant fragment in XbaI-digested genomic DNA; the 5′ probe hybridized to a 13.4-kb wild-type or 8.4-kb recombinant fragment in EcoRI digestions. Homologous recombinant clones were confirmed by independent XbaI and EcoRI digestions and hybridization with 3′ and 5′ probes, respectively. Additionally, the genomic sequence of exon 8 was verified by sequencing, and a Neo probe was used to exclude random integration events in the selected clones.

### Generation of knock-in mice

Mutant ES cells were injected into Balb/c blastocysts, and one recombinant ES clones was found to be capable of germline transmission. The *Neo*R gene flanking the *Zap70*^YEEI^ mutant locus was removed by crossing chimeric mice with *Cre*-expressing deleter mice (19). Mice carrying the deletion of the loxP-flanked *NeoR* gene were subsequently intercrossed to produce homozygous mutant offspring. Screening of the intended ZAP-70 mutations mice for the presence of the intended ZAP-70 mutations was performed by PCR using the following oligonucleotides primers: YEEI-Fw (5’-TGTGTGCTGGATGTAGGACCCAGG-3’) and YEEI-Rv (5’-TGGGGAAAGCCACTGCTGGATGTC-3’). All analyses were performed after backcrossing the mutant mice onto a C57BL/6 background for at least 7 generations.

### Preparation of cell lysates and western blotting

Thymocytes from wild-type or mutant mice were isolated and incubated with 5 μg/ml biotinylated anti-CD3 mAb for 30 min on ice, followed by stimulation at 37 °C with 10 μg/ml streptavidin. Cells were then lysed at a density of 5 × 10^7^ cells/ml in 2X Laemmli buffer, followed by incubation at 95 °C for 5 min and sonication. For Western blotting, whole-cell lysates were resolved by SDS-PAGE and transferred to PVDF membranes. Membranes were blocked and incubated with the indicated primary antibodies, followed by appropriate secondary antibodies conjugated to either IRDye 800CW (Li-Cor, Lincoln, NE, USA) or horseradish peroxidase (HRP). Reactive proteins were visualized using the Odyssey CLx Infrared Imaging System (Li-Cor, Lincoln, NE, USA) or via enhanced chemiluminescence (ECL) using a ChemiDoc Touch Imaging System (Bio-Rad Laboratories, Hercules, CA, USA). For reprobing, membranes were incubated for 10 min at room temperature with WB Stripping Solution (Nacalai Tesque, Kyoto, Japan) and washed with TTBS. Protein bands were quantified by densitometric analysis.

### Activation and proliferation assays

Splenocytes were isolated, and T lymphocytes were purified via negative selection using the MojoSort™ Mouse CD3 T Cell Isolation Kit (Biolegend, San Diego, CA, USA). Purified T cells were labeled with CellTrace Violet™ for 20 min at 37°C. After staining, cells were resuspended at 3.0 × 10^6^ cells/ml in RPMI1640 medium supplemented with 10% fetal bovine serum, 1 mM sodium pyruvate, 50 μM β-mercaptoethanol, and penicillin-streptomycin. Cells were seeded in 48-wells plates (1 × 10^6^ cells/well) and stimulated with anti-CD3/CD28-coupled microbeads at two ratios: 8 × 10^4^ beads (1:12.5 bead-to-cell ratio) and 2 × 10^5^ beads (1: 5 ratio). Unstimulated cells were included as a control. After 24 hours of stimulation, 100 μl of the cell suspension was collected and viable cells (Annexin-V negative) were analyzed via flow cytometry for the expression of CD4, CD8 and CD69. CD4 and CD8 T-cell proliferation was assessed after 72 h on the remaining culture by flow cytometry.

### Statistics

Statistics were performed with GraphPad Prism using one-way or two-way ANOVA tests. Levels of significance p < 0.05 are presented as *, p < 0.01 as **, p < 0.001 as *** and p < 0.0001 as ****.

## Data Availability

The original contributions presented in the study are included in the article/**Supplementary Material**. Further inquiries can be directed to the corresponding authors.
