# Supplementary material for: A Zap70 gain-of-function mutation alters mouse thymic maturation
Source: Front Immunol. 2026 Oct 6;17:1932213. doi: 10.3389/fimmu.2026.1932213 (PMC13680439; doi:10.3389/fimmu.2026.1932213)
Supplement: Supplementary file 1 [file Supplementaryfile1.pdf]

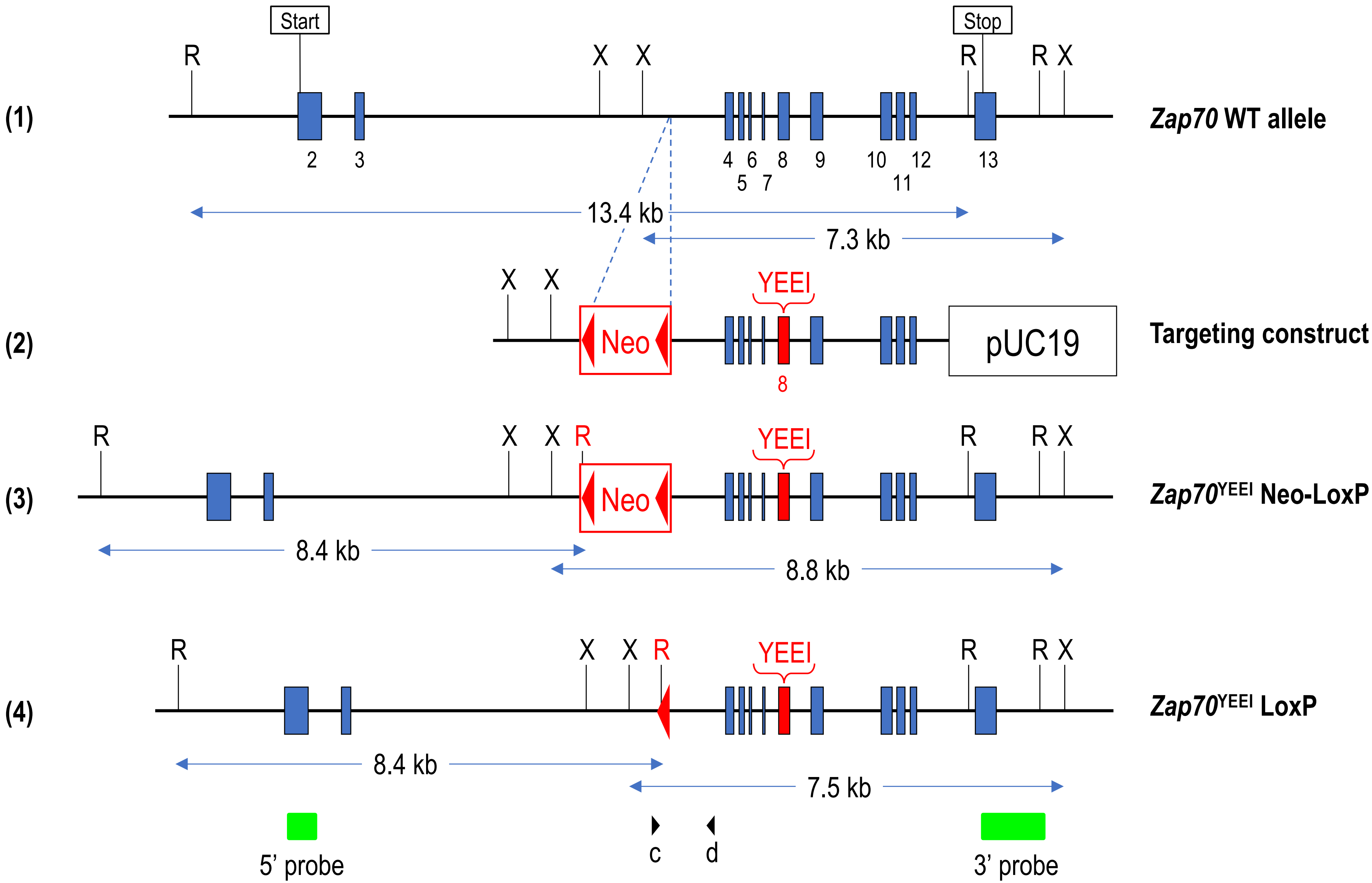

Supplementary Figure 1

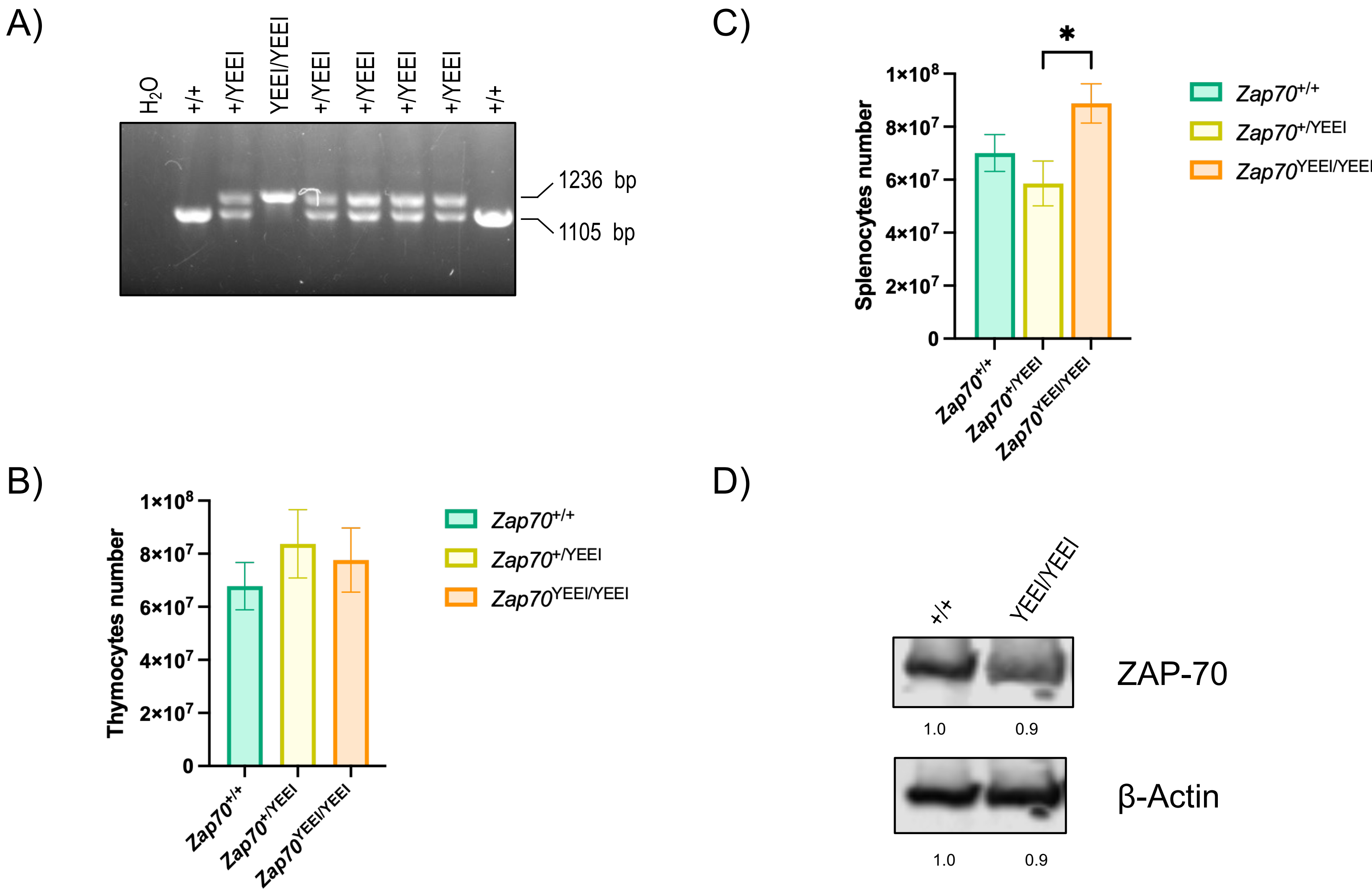

Supplementary Figure 2

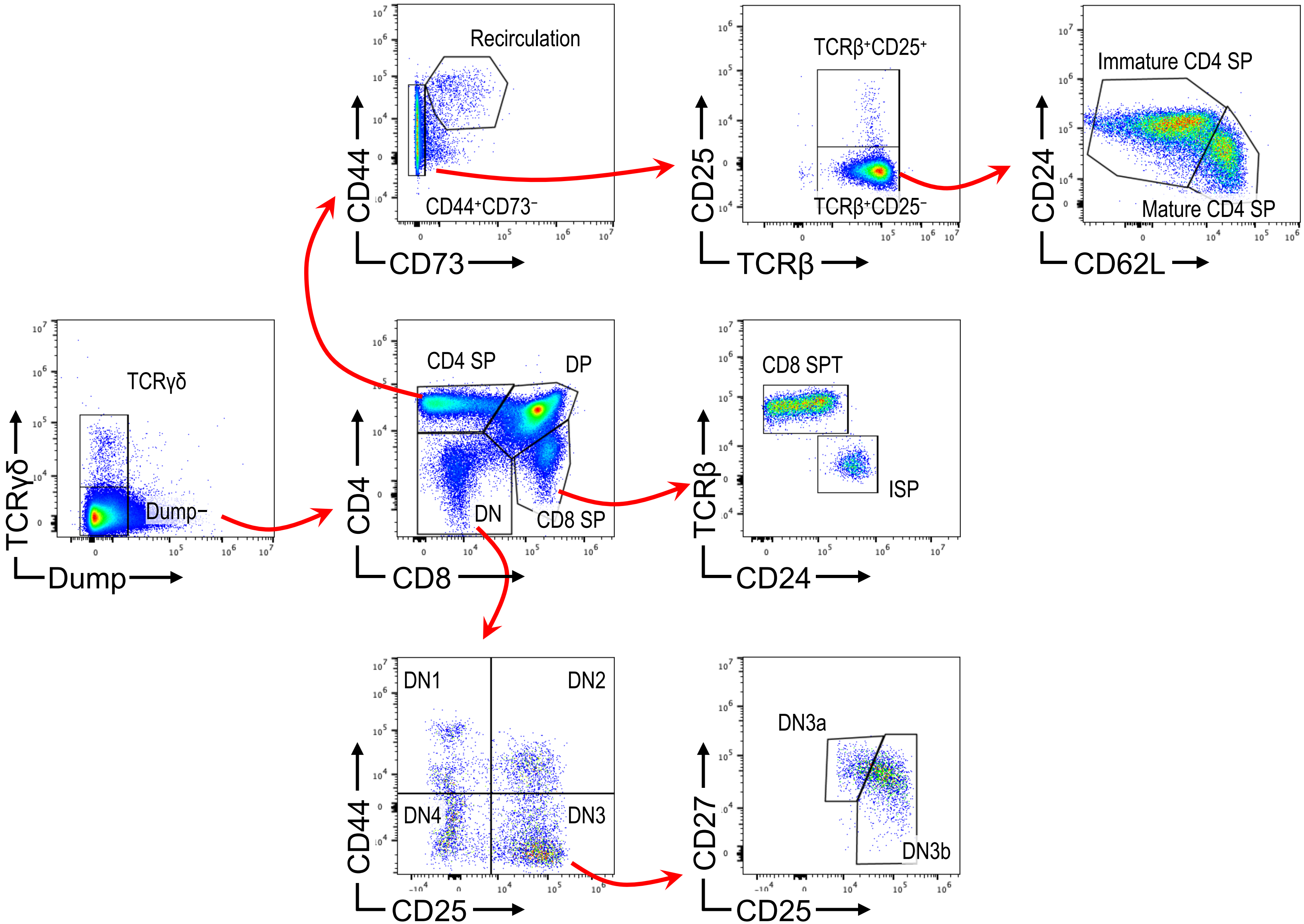

Supplementary Figure 3

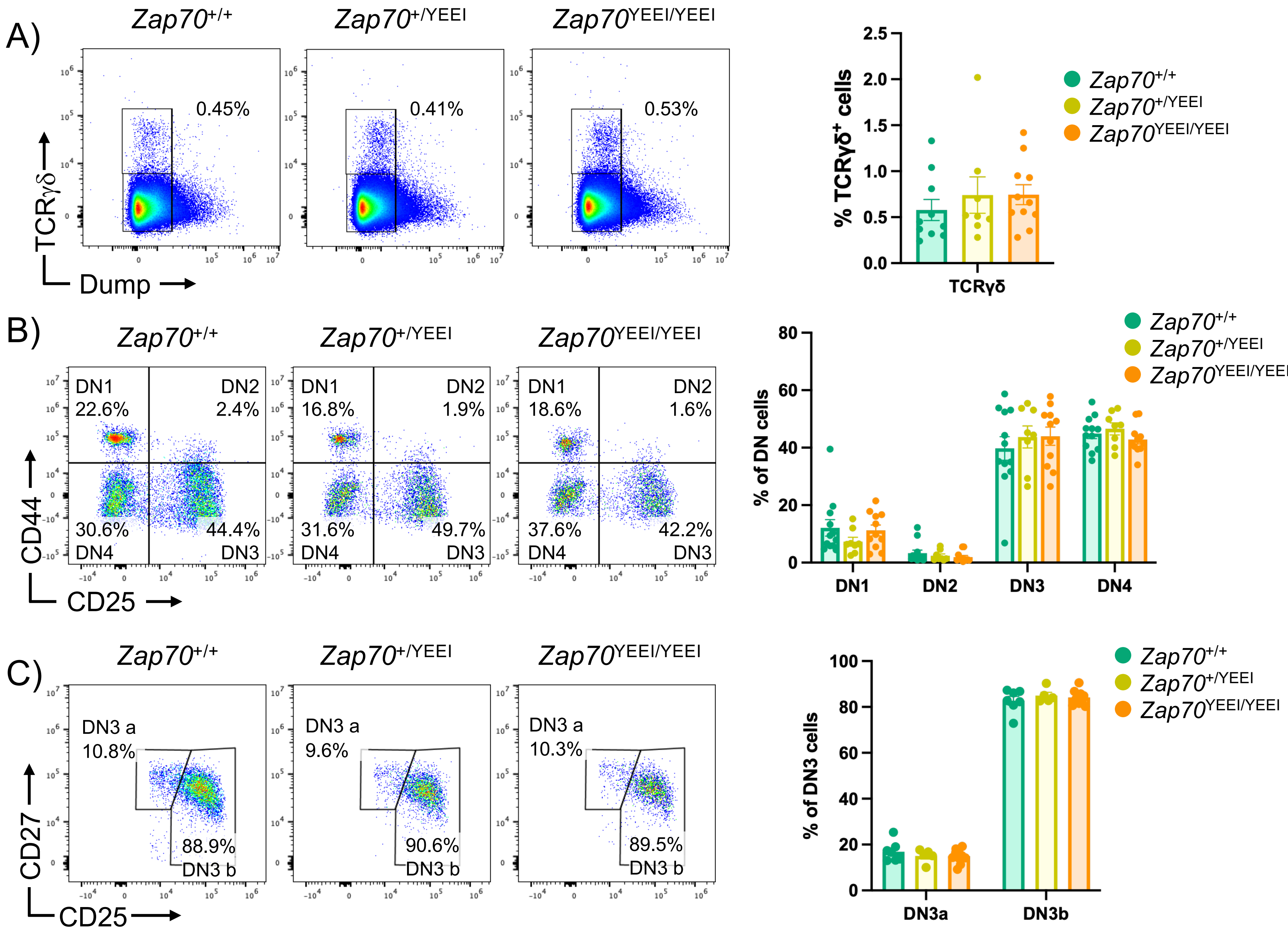

Supplementary Figure 4

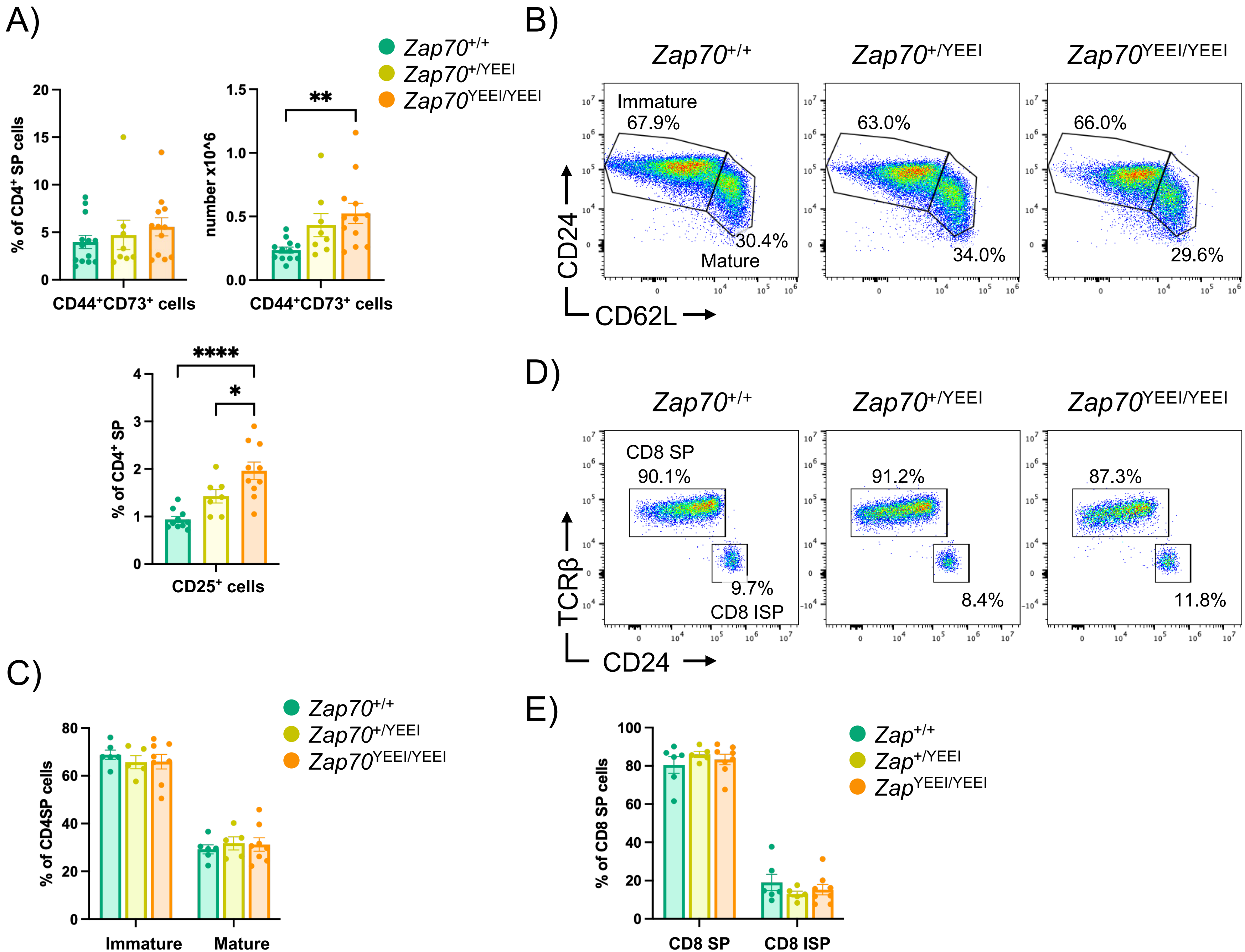

Supplementary Figure 5

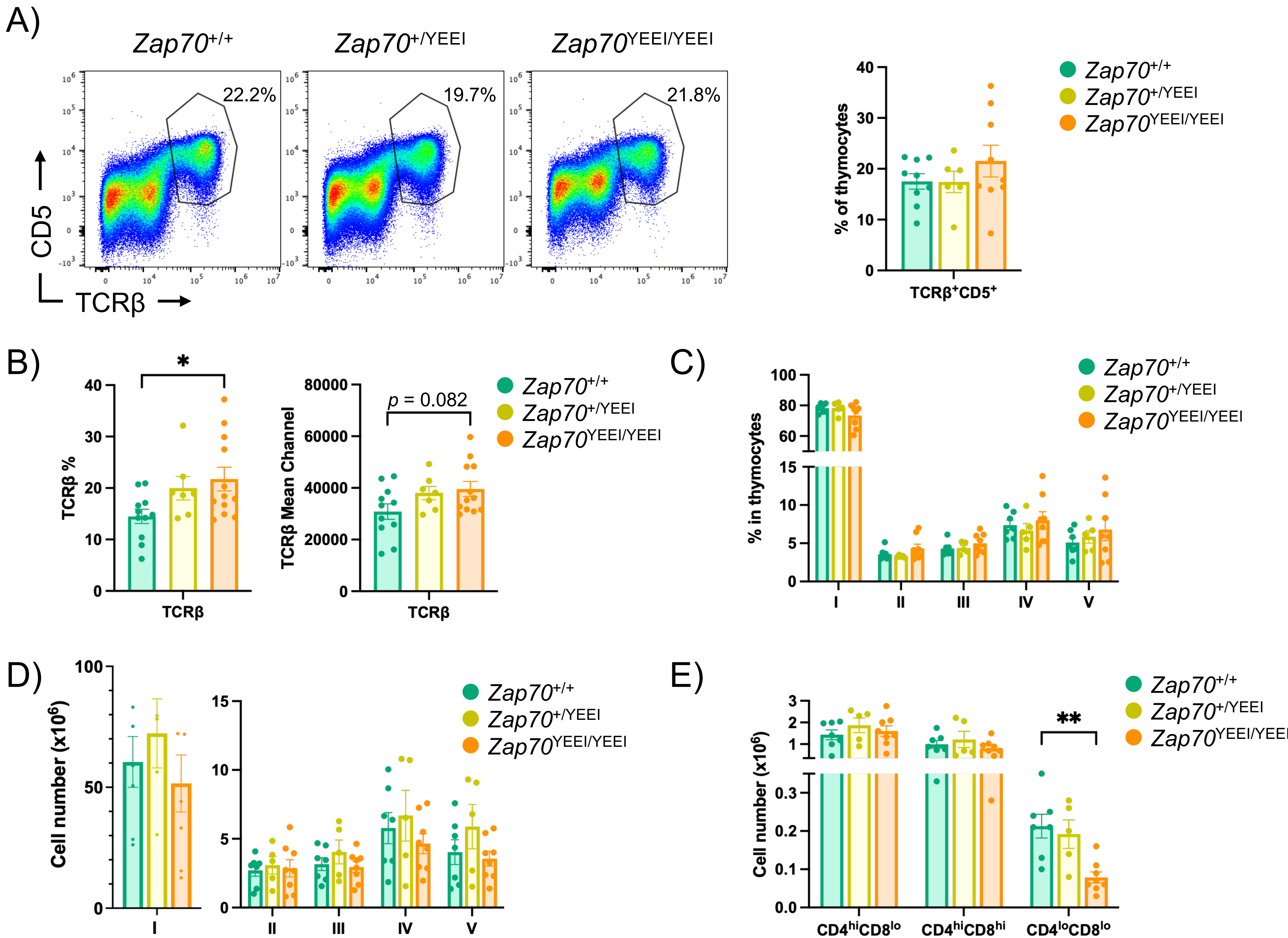

Supplementary Figure 6

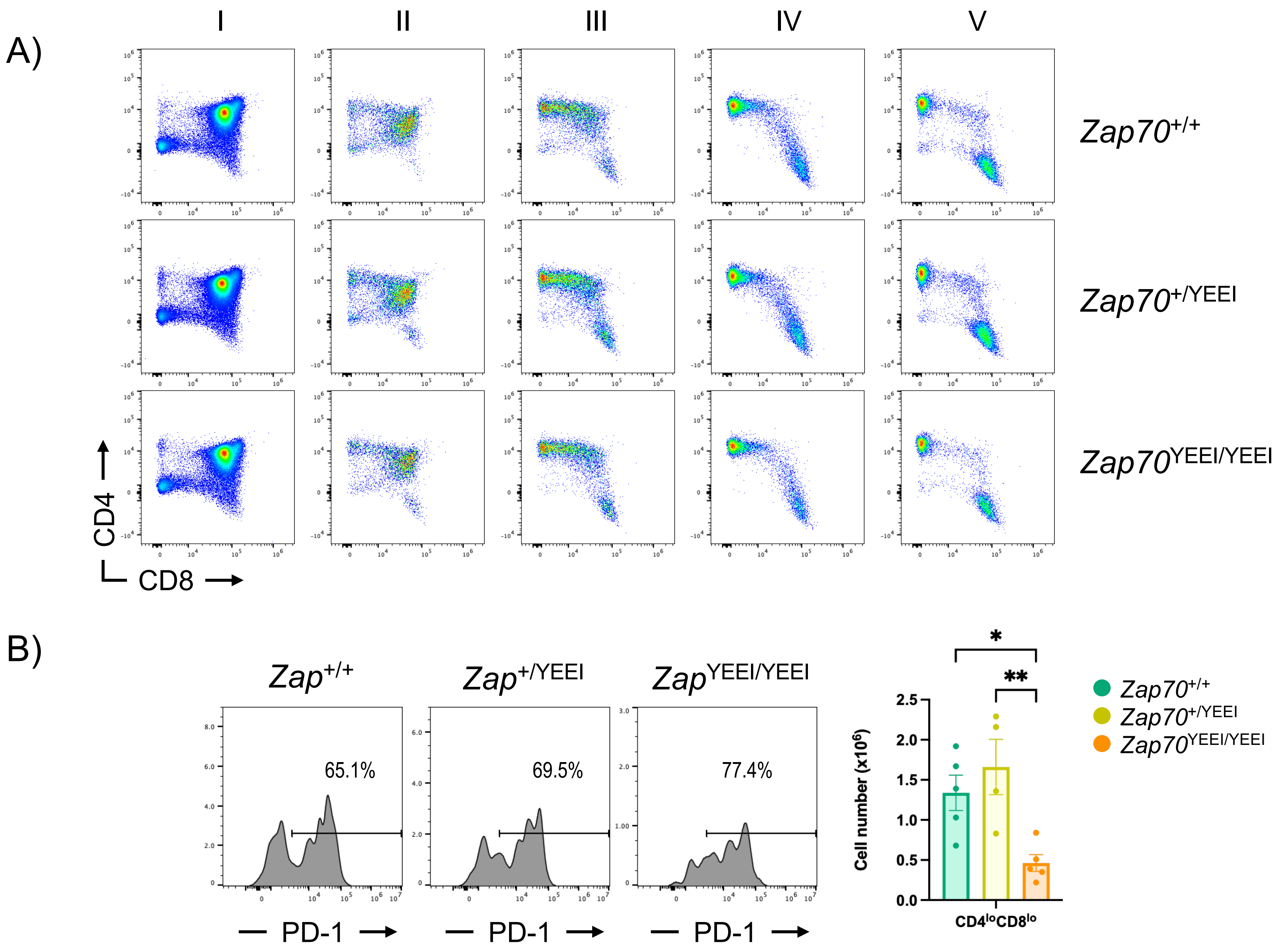

Supplementary Figure 7

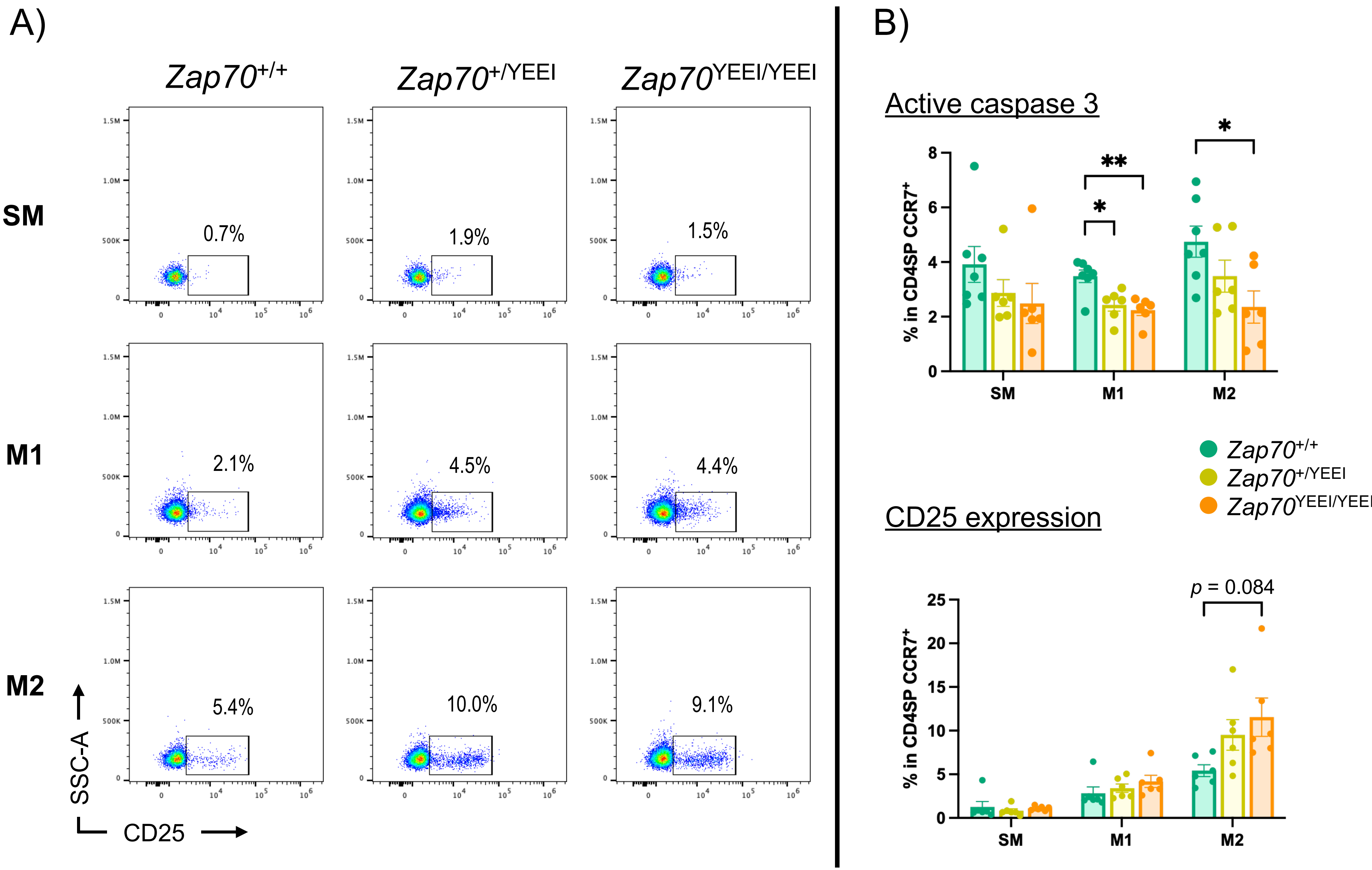

Supplementary Figure 8

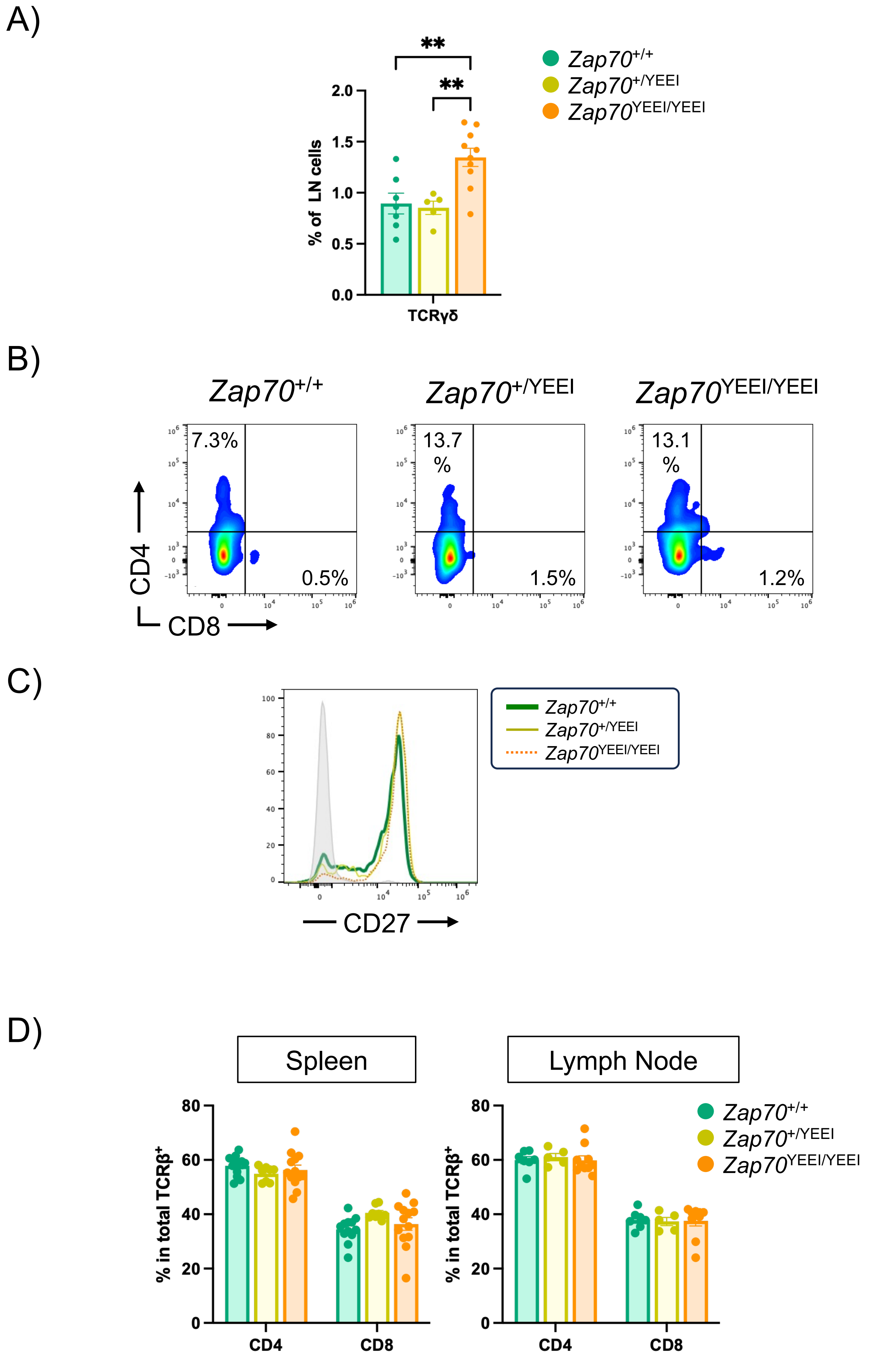

Supplementary Figure 9

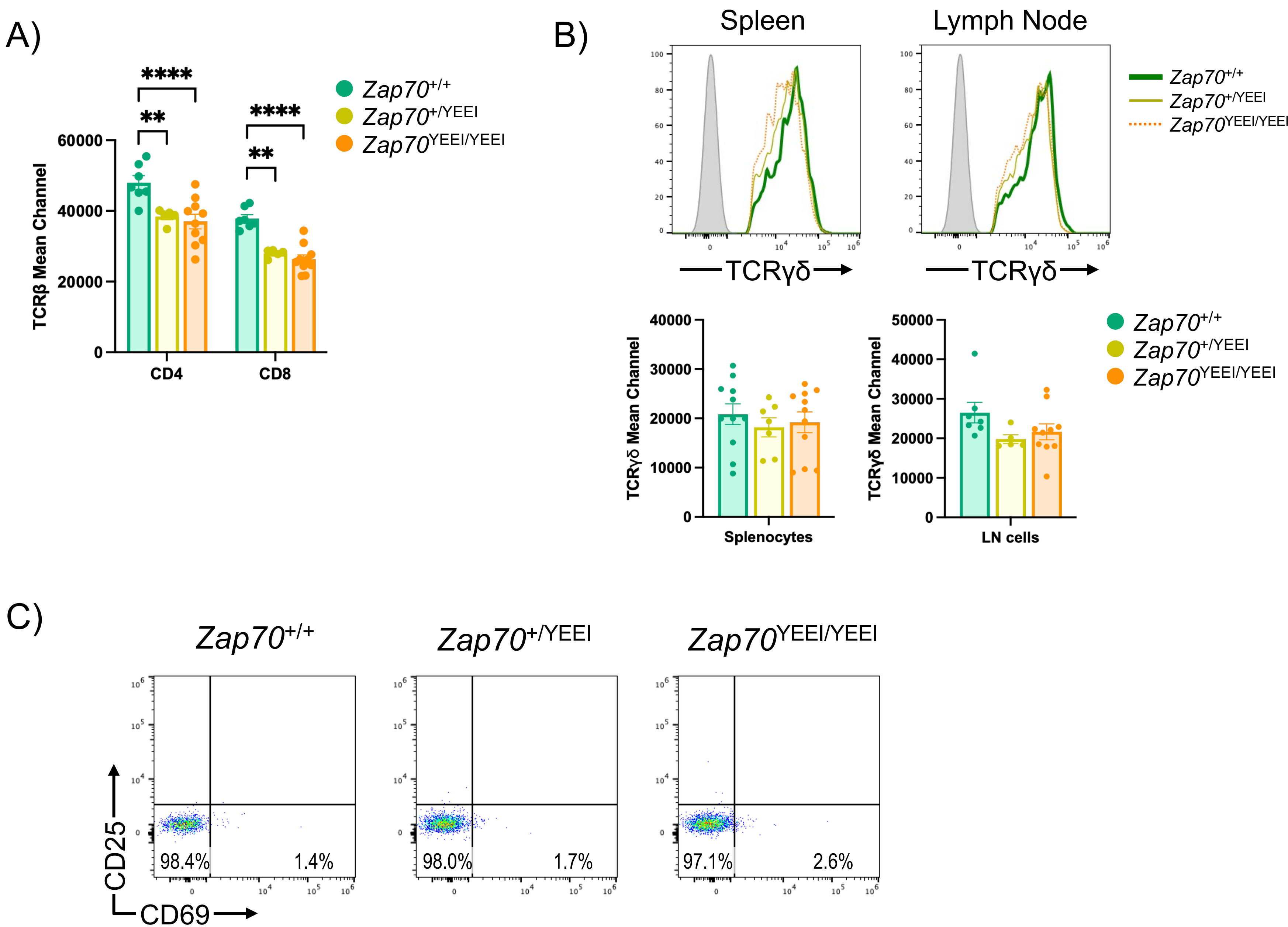

Supplementary Figure 10

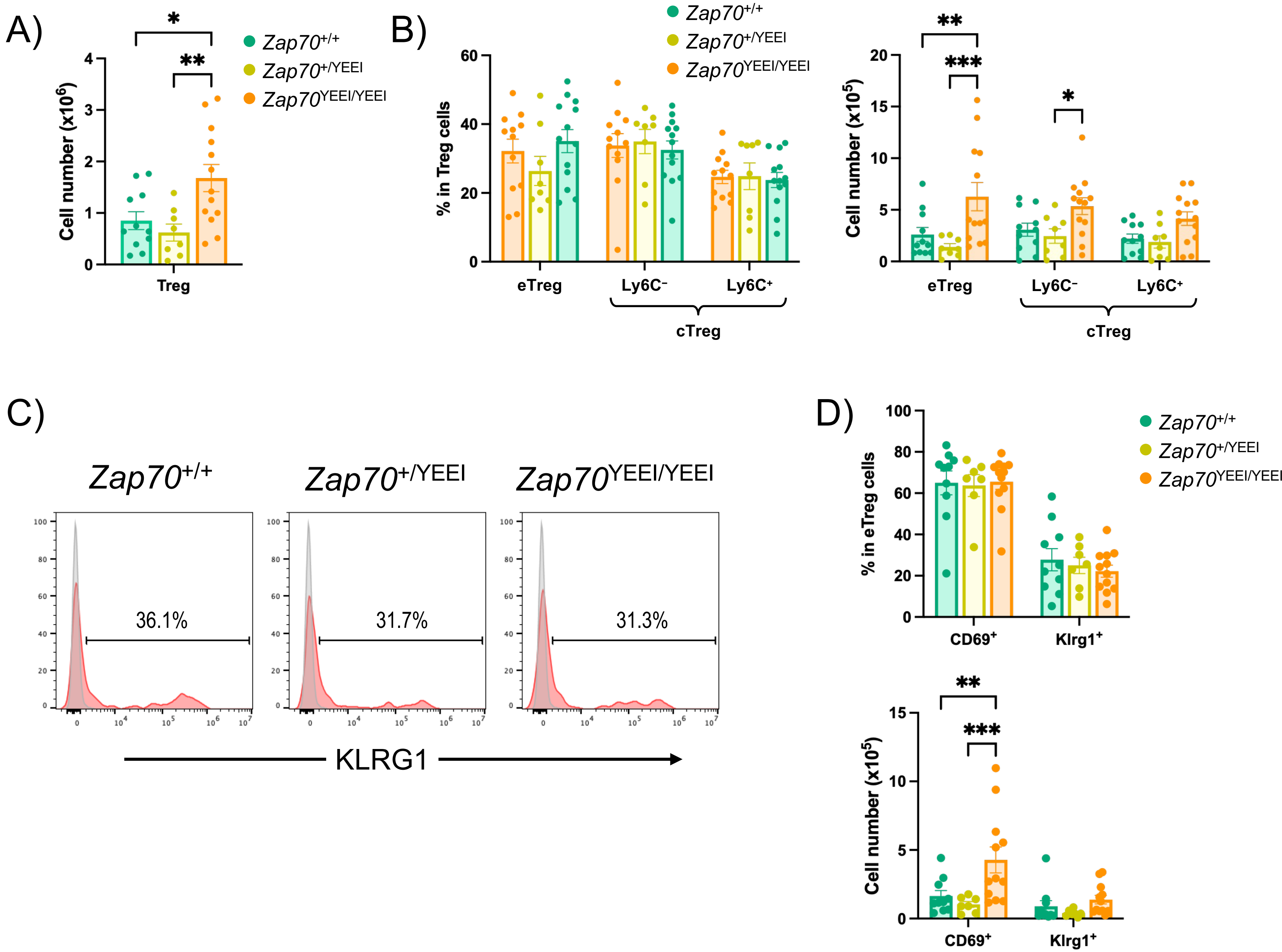

Supplementary Figure 11

Supplementary Figure legends

Supplementary Figure 1. Generation and identification of *Zap70*^{YEEI} mice. (1) Partial restriction map of the wild-type *Zap70* gene. Exons 2–13 are depicted as blue boxes. Restriction sites are indicated as R (EcoRI) and X (XbaI). The start and stop codons, located in exons 2 and 13 respectively, are shown. (2) Targeting vector used to introduce the *Zap*^{YEEI} mutation. The codons in exon 8 encoding Y^{319}SDP were replaced with a sequence encoding Y^{319}EEI. The targeting construct contains a loxP-flanked NeoR cassette inserted in the intron upstream of exon 4. (3) Structure of the targeted locus. (4) Structure of the targeted locus following Cre-mediated excision of the NeoR cassette. The 5' and 3' probes used for Southern blot analysis are shown as green boxes. The position of PCR primers (c and d) used to monitor transmission of the *Zap70*^{YEEI} mutation are indicated by black triangles.

Supplementary Figure 2. Identification of *Zap70*^{YEEI} mice. (A) DNA-PCR analysis of wild-type (+/w), heterozygous (+/YEEI), and homozygous (YEEI/YEEI) littermates using the pair of primers shown in Supplementary Figure 1. The single Lox P site remaining in the modified *Zap70*^{YEEI} allele after deletion of the NeoR cassette resulted in PCR product 131 bp-longer than the one amplified from the wild-type allele. The amplified products were run on an agarose gel and stained with ethidium Red Safe. (B) Bar graphs representing the number of thymocytes in wild-type (*Zap70*$^{+/+}$, n = 12), heterozygous (*Zap70*$^{+/YEEI}$, n = 8), and homozygous (*Zap70*$^{YEEI/YEEI}$, n = 13) mutant mice aged from 6 to 16 weeks. Brackets on each bar represent the standard error mean. (C) Bar graphs representing the number of total splenocytes in wild-type (n = 12), heterozygous (n = 8), and homozygous (n = 13) mutant mice aged from 6 to 16 weeks. Error bars represent the standard error of the mean. Asterisks indicate statistical significance (* $p < 0.05$). (D) Western blot analysis for the expression of ZAP-70 and β-actin in lysates from 2 x 10^6 fresh thymocytes of wild-type (+/+) and homozygous (YEEI/YEEI) mutant mice. Numbers below the blots indicate relative densitometric quantification normalized to wild-type controls.

Supplementary Figure 3. Flow cytometry gating strategy for the analysis of thymic populations. Living thymocytes were first identified based on forward- and side-scatter properties and exclusion of Live/Dead Yellow–positive cells. From the viable population, TCRγδ$^+$ cells and MHC-II$^+$, CD11b$^+$, and NK1.1$^+$ (Dump) cells were excluded to eliminate non-conventional or contaminating lineages. Subsequent gates were applied to define conventional T-cell subsets for downstream phenotypic analyses.

Supplementary Figure 4. Effects of *Zap70*^{YEEI} mutation on thymic development. (A) Viable thymocytes from 6- to 23-week-old adult wild-type (*Zap70*$^{+/+}$), heterozygous (*Zap70*$^{+/YEEI}$), and homozygous (*Zap70*$^{YEEI/YEEI}$) mutant mice were analyzed for TCRγδ expression. MHC-II$^+$, CD11b$^+$, and NK1.1$^+$ (Dump) cells were excluded from the analysis. The bar graphs on the right show the mean percentage of TCRγδ$^+$ thymocytes in wild-type (n = 10), heterozygous (n = 8), and homozygous (n = 13) mice aged from 6-16 weeks. Error bars represent the standard error of the mean. **(B)** DN thymocytes (gated on Dump$^-$CD4$^-$CD8$^-$ cells) from 6-16-week-old mice were analyzed for CD44 and CD25 expression. Percentages of cells in each quadrant are indicated. The bar graph on the right summarizes the percentage of cells within each indicated compartment for wild-type (n = 12), heterozygous (n = 8), and homozygous (n = 11) mice. **(C)** DN3a and DN3b subpopulations were analyzed by CD25 and CD27 staining of DN3 thymocytes from *Zap70*$^{+/+}$ (n =7), *Zap70*$^{+/YEEI}$ (n = 5), and *Zap70*$^{YEEI/YEEI}$ (n = 10) mice. The bar graph on the right shows that no significant differences were observed among the three genotypes.

Supplementary Figure 5. Impact of the *Zap70*^{YEEI} mutation on thymic development. (A) Bar graphs showing the mean percentages (top-left) and total numbers (top-right) of CD44$^+$CD73$^+$ recirculating CD4SP cells in the thymus of wild-type (*Zap70*$^{+/+}$, n = 12),

heterozygous (*Zap70*$^{+/YEEI}$, n = 8), and homozygous (*Zap70*$^{YEEI/YEEI}$, n= 12) mutant mice, aged from 6 to 16 weeks. Bottom panel shows the percentage of $CD25^{+}$ cells among $CD44^{+}CD73^{-}$ CD4SP thymocytes from wild-type (n = 10), heterozygous (n = 7), and homozygous (n= 10) mutant mice, aged from 6 to 16 weeks. Asterisks indicate statistical significance (* p < 0.05; ** p<0.01; **** p<0.0001). **(B)** Dot plots showing CD62L and CD24 expression in non-recirculating ($CD44^{+}CD73^{-}$) $TCR\beta^{+}CD25^{-}$ cells. **(C)** Bar graphs showing the mean percentages of immature ($CD24^{hi}CD62L^{lo}$) and mature ($CD24^{lo}CD62L^{hi}$) CD4 single positive cells, gated on the $TCR\beta^{+}CD25^{-}$ compartment (as shown in Supplementary Figure 3), in wild-type (n = 6), heterozygous (n = 5), and homozygous (n = 8) mutant mice aged from 6-16 weeks. Error bars represent the standard error of the mean. **(D)** Analysis of CD8 intermediate single-positive using the strategy shown in Supplementary Figure 3. One representative experiment is shown. **(E)** Bar graphs showing the mean percentages of bona fide CD8 single-positive cells ($TCR\beta^{+}CD24^{lo}$) and intermediate CD8 single-positive cells (ISP) ($TCR\beta^{lo}CD24^{hi}$) in wild-type (n = 6), heterozygous (n = 5), and homozygous (n = 8) mice aged from 6-16 weeks.

Supplementary Figure 6. Analysis of positive selection in the thymus of *Zap70*^{YEEI} mutant mice. (A) Analysis of TCRβ and CD5 expression of Dump negative thymocytes (see Suppl. Fig. 3) from wild-type (*Zap70*$^{+/+}$), heterozygous (*Zap70*$^{+/YEEI}$), and homozygous (*Zap70*$^{YEEI/YEEI}$) mutant mice. The gate shown in the dot plots represent "signaled" thymocytes that can be cells that have undergone, or are currently undergoing, positive selection. The bar graphs on the right show the mean percentage of $TCR\beta^{high}CD5^{+}$ thymocytes in wild-type (n = 9), heterozygous (n = 6), and homozygous (n = 9) mutant mice aged from 6-16 weeks. Error bars represent the standard error of the mean. **(B)** Bar graphs showing the mean percentages of $TCR\beta^{+}$ (left panel) and the intensity of TCRβ in Dump negative thymocytes (right panel) from wild-type (n = 11), heterozygous (n = 7), and homozygous (n = 12) mutant mice aged from 6-16 weeks. Asterisk indicates statistical significance (* p < 0.05). **(C)** Percentage of thymocytes in each developmental stages defined by TCR vs. CD69 expression in wild-type (n = 7), heterozygous (n = 5), and homozygous (n = 8) mutant mice aged from 6-16 weeks. **(D)** Thymocyte number in each developmental stage. **(E)** Bar graphs representing the number of thymocytes in subpopulations of stage III in wild-type (n = 7), heterozygous (n = 5), and homozygous (n = 8) mutant mice aged from 6-16 weeks. Asterisks indicate statistical significance (** p < 0.01).

Supplementary Figure 7. Stage III thymocytes analysis. (A) CD4 and CD8 profile in each thymocyte developmental stage defined by TCRβ versus CD69 expression. **(B)** Histograms showing the flow cytometric analysis of PD-1 expression in $CD4^{lo}CD8^{lo}$ stage III thymocytes (left panels). The bar graphs on the right show the number of $CD4^{lo}CD8^{lo}PD\text{-}1^{+}$ thymocytes in wild-type (n = 5), heterozygous (n = 4), and homozygous (n = 5) mutant mice aged 6-16 weeks. Brackets on each bar represent the standard error of the mean. Asterisks indicate statistical significance (* p < 0.05; ** p < 0.01).

Supplementary Figure 8. Analysis of final steps of CD4 SP thymic development. (A) Flow cytometric analysis of CD25 expression by semi-mature (SM), mature 1 (M1), and mature 2 (M2) CD4 SP medullary thymocytes from adult wild-type (*Zap70*$^{+/+}$), heterozygous (*Zap70*$^{+/YEEI}$), and homozygous (*Zap70*$^{YEEI/YEEI}$) mutant mice. One representative experiment is shown. **(B)** Bar graphs showing the mean percentages of SM, M1, and M2 cells expressing active caspase 3 (upper panel) and CD25 (lower panel). Data were pooled from the analysis of wild-type (n = 6), heterozygous (n = 6), and homozygous (n = 6) mice aged 6-16 weeks. Error bars represent the standard error of the mean. Asterisks indicate statistical significance (* p < 0.05; ** p < 0.01).

Supplementary Figure 9. Characterization of peripheral lymphocytes. (A) Bar graph showing the average of the percentage of $TCR\gamma\delta^{+}$ cells in the lymph nodes from wild-type

($Zap70^{+/+}$, n = 7), heterozygous ($Zap70^{+/YEEI}$, n = 5), and homozygous ($Zap70^{YEEI/YEEI}$, n = 10) mice aged from 6-16 weeks. Error bars represent the standard error of the mean. Asterisks indicate statistical significance (** p<0.01). (B) Dot plots showing CD4 vs. CD8 expression in TCRγδ$^+$ lymphocytes from the spleens of wild-type, heterozygous, and homozygous mutant mice. (C) CD27 expression intensity analyzed by flow cytometry in TCRγδ$^+$ cells from the spleens of wild-type (green thick line), heterozygous (yellow thin line), and homozygous (dotted orange line) mutant mice. The grey shadowed line represents the isotype control. (D) Bar graphs showing the mean percentages of CD4 and CD8 expression in TCRβ$^+$ lymphocytes obtained from the spleens or lymph nodes of wild-type, heterozygous, and homozygous mutant mice. For splenocytes, 12 wild-type mice, 8 heterozygous mice, and 13 homozygous mice aged from 6-16 weeks were analyzed. For lymph nodes, 7 wild-type mice, 5 heterozygous mice, and 10 homozygous mice were analyzed. Error bars represent the standard error of the mean.

Supplementary Figure 10. Phenotypic analysis of peripheral lymphocytes. (A) Bar graphs representing the average values of the mean channel for TCRβ expression in CD4$^+$ and CD8$^+$ lymphocytes from the lymph nodes of wild-type ($Zap70^{+/+}$, n = 7), heterozygous ($Zap70^{+/YEEI}$, n = 5), and homozygous ($Zap70^{YEEI/YEEI}$, n = 12) mutant mice aged 6-16 weeks. Error bars represent the standard error of the mean. Asterisks indicate statistical significance (** p<0.01; **** p<0.0001). **(B)** TCRγδ expression intensity analyzed by flow cytometry in TCRγδ$^+$ lymphocytes from the spleens or lymph nodes of wild-type (green thick line), heterozygous (yellow thin line), and homozygous (dotted orange line) mutant mice (top panels). The grey shadowed line represents the isotype control. The lower bar graphs show the average values of the mean channel for TCRγδ expression in splenocytes (left panel) or lymph node cells (right panel). For splenocytes, 11 wild-type mice, 7 heterozygous mice, and 11 homozygous mice were analyzed. For lymph nodes, 7 wild-type mice, 5 heterozygous mice, and 10 homozygous mice were analyzed. **(C)** Dot plots showing the expression of CD69 and CD25 by γδ T-cells from the spleens of wild-type, heterozygous and homozygous mutant mice. One representative experiment is shown.

Supplementary Figure 11. Characterization of the splenic Treg cell compartment in *Zap*^{YEEI} mutant mice. (A) Bar graph showing the average number of Treg cells (TCRβ$^+$CD4$^+$CD25$^+$) in wild-type ($Zap^{+/+}$, n = 11), heterozygous ($Zap^{+/YEEI}$, n = 8), and homozygous ($Zap^{YEEI/YEEI}$, n = 13) mutant mice aged 6-16 weeks. Brackets on each bar represent the standard error mean. **(B)** Bar graphs showing the average percentage (left panel) and number (right panel) of the different subpopulations of Treg cells defined by the expression of Ly6C and CD62L. Splenocytes from wild-type (n= 11), heterozygous (n = 8) and homozygous (n = 13) mutant mice aged 6-16 weeks were analyzed. **(C)** Histograms showing KLRG1 expression in eTreg (Ly6C$^-$CD62L$^-$) cells from the spleens of $Zap^{+/+}$, $Zap^{+/YEEI}$, and $Zap^{YEEI/YEEI}$ mice. One representative experiment is shown. **(D)** Bar graphs show respectively the percentage (upper panel) and total numbers (lower panel) of CD69$^+$ and KLRG1$^-$ splenocytes from $Zap^{+/+}$ (n = 10), $Zap^{+/YEEI}$ (n = 7), and $Zap^{YEEI/YEEI}$ (n = 12) mice. Brackets on each bar represent the standard error mean. Asterisks indicate statistical significance (* p<0.05; ** p<0.01; **** p<0.0001).
