# Supplementary material for: A Zap70 gain-of-function mutation alters mouse thymic maturation
Source: Front Immunol. 2026 Oct 6;17:1932213. doi: 10.3389/fimmu.2026.1932213 (PMC13680439; doi:10.3389/fimmu.2026.1932213)
Supplement: Supplementary file 2 [file Supplementaryfile2.pdf]

Supplementary Table 1. Sex, age, and total thymocyte counts of individual mice analyzed in Supplementary Figure 2.

Mouse ID	Genotype	Sex	Age (Weeks)	Total Thymocytes ($x10^6$)
WT-001	+/+	Male	11	62,25
Ho-001	YEEI/YEEI	Male	12	90,25
Ho-002	YEEI/YEEI	Male	13	56,25
WT-002	+/+	Male	16	62,50
Ho-011	YEEI/YEEI	Female	15	74,75
Ho-012	YEEI/YEEI	Female	15	56,50
WT-013	+/+	Female	6	101,75
He-006	+/YEEI	Female	6	109,25
Ho-020	YEEI/YEEI	Female	6	92,25
WT-014	+/+	Male	7	102,00
He-007	+/YEEI	Male	7	150,50
Ho-021	YEEI/YEEI	Male	6	48,25
WT-015	+/+	Female	7	125,50
He-008	+/YEEI	Female	7	68,75
Ho-022	YEEI/YEEI	Female	7	137,00
WT-016	+/+	Female	9	77,50
He-009	+/YEEI	Female	9	101,25
Ho-023	YEEI/YEEI	Female	9	66,50
WT-017	+/+	Male	12	33,25
He-010	+/YEEI	Male	12	37,50
Ho-024	YEEI/YEEI	Male	12	24,00
Ho-025	YEEI/YEEI	Male	16	20,75
WT-020	+/+	Female	10	49,90
WT-021	+/+	Male	16	85,25
WT-023	+/+	Male	10	30,50
He-018	+/YEEI	Male	8	88,25
Ho-029	YEEI/YEEI	Male	9	162,50
WT-024	+/+	Male	13	45,00
He-019	+/YEEI	Male	11	57,50
Ho-030	YEEI/YEEI	Male	13	48,25
WT-025	+/+	Male	16	37,50
He-020	+/YEEI	Female	14	56,75
Ho-030	YEEI/YEEI	Female	16	131,00

Supplementary Table 1. Sex, age, and total thymocyte cell counts of individual mice. Metadata and total thymocyte counts (× 10^6) for all individual mice (N = 33; 6–16 weeks of age) analyzed in Supplementary Figure 2, listed by Mouse ID, genotype (wild-type, +/+; heterozygous, +/YEEI; homozygous, YEEI/YEEI), sex, and exact age in weeks.
